# Neural complexity is a common denominator of human consciousness across diverse regimes of cortical dynamics

**DOI:** 10.1038/s42003-022-04331-7

**Published:** 2022-12-15

**Authors:** Joel Frohlich, Jeffrey N. Chiang, Pedro A. M. Mediano, Mark Nespeca, Vidya Saravanapandian, Daniel Toker, John Dell’Italia, Joerg F. Hipp, Shafali S. Jeste, Catherine J. Chu, Lynne M. Bird, Martin M. Monti

**Affiliations:** 1 Department of Psychology, University of California Los Angeles, 90095 Pritzker Hall, Los Angeles, CA USA; 2 Department of Computational Medicine, University of California Los Angeles, Los Angeles, CA USA; 3 Department of Computing, Imperial College London, London, UK; 4 Department of Psychology, University of Cambridge, Cambridge, UK; 5 Department of Neurosciences, University of California San Diego, San Diego, CA USA; 6 Department of Neurology, Rady Children’s Hospital San Diego, San Diego, CA USA; 7 Center for Autism Research and Treatment, University of California Los Angeles, Semel Institute for Neuroscience, Los Angeles, CA USA; 8 Institute for Advanced Consciousness Studies, Santa Monica, CA USA; 9 Roche Pharma Research and Early Development, Neuroscience and Rare Diseases, Roche Innovation Center Basel, Basel, Switzerland; 10 Department of Neurology, Massachusetts General Hospital, Harvard Medical School, Boston, MA USA; 11 Department of Pediatrics, University of California San Diego, San Diego, CA USA; 12 Division of Genetics/Dysmorphology, Rady Children’s Hospital - San Diego, San Diego, CA USA; 13 Deptment of Neurosurgery, UCLA Brain Injury Research Center, David Geffen School of Medicine, University of California Los Angeles, Los Angeles, CA USA; 14 Present Address: Institute for Neuromodulation and Neurotechnology, University Hospital and University of Tuebingen, Tuebingen, Germany; 15 Present Address: Children’s Hospital Los Angeles, Los Angeles, CA USA

**Keywords:** Diagnostic markers, Circadian rhythms and sleep, Disorders of consciousness, Psychology

## Abstract

What is the common denominator of consciousness across divergent regimes of cortical dynamics? Does consciousness show itself in decibels or in bits? To address these questions, we introduce a testbed for evaluating electroencephalogram (EEG) biomarkers of consciousness using dissociations between neural oscillations and consciousness caused by rare genetic disorders. Children with Angelman syndrome (AS) exhibit sleep-like neural dynamics during wakefulness. Conversely, children with duplication 15q11.2-13.1 syndrome (Dup15q) exhibit wake-like neural dynamics during non-rapid eye movement (NREM) sleep. To identify highly generalizable biomarkers of consciousness, we trained regularized logistic regression classifiers on EEG data from wakefulness and NREM sleep in children with AS using both entropy measures of neural complexity and spectral (i.e., neural oscillatory) EEG features. For each set of features, we then validated these classifiers using EEG from neurotypical (NT) children and abnormal EEGs from children with Dup15q. Our results show that the classification performance of entropy-based EEG biomarkers of conscious state is not upper-bounded by that of spectral EEG features, which are outperformed by entropy features. Entropy-based biomarkers of consciousness may thus be highly adaptable and should be investigated further in situations where spectral EEG features have shown limited success, such as detecting covert consciousness or anesthesia awareness.

## Abstract (web summary)

EEG measurements in children with Angelman or duplication 15q11.2-13.1 syndrome reveal a dissociation between consciousness and sleep-like spectral content, with complexity-based measures superseding other markers of consciousness.

## Introduction

EEG is an attractive modality for obtaining readouts of cortical activity and conscious state, as it is a direct, non-invasive measure of neural activity that is inexpensive and easily deployed at the bedside or in the operating room. Many EEG features have been identified as candidate biomarkers of conscious state^1,2^. In spontaneous EEG, features of interest include: (1) spectral features, such as delta (1–4 Hz) power^3,4^ and (2) entropy or “complexity” estimates such as Lempel-Ziv complexity (LZ)^5–7^. Furthermore, both spectral and entropy measures can reflect activity at single channels (SC) or statistical dependencies, i.e., functional connectivity (FC), between channels. When EEG recordings are highly abnormal, it remains unclear whether the classification performance of entropy features is upper-bounded by spectral features, or if entropy might convey additional information.

By utilizing EEG data recorded from patients in the vegetative state (also known as unresponsive wakefulness syndrome) or minimally conscious state, as well as EEG data recorded from healthy volunteers in non-rapid eye movement (NREM) sleep or under anesthesia, prior studies^3,7,8^ have described EEG biomarkers of consciousness based on both spectral and complexity/entropy features. These approaches are informative and well suited to discovering EEG features that contrast conscious and unconscious states within a particular clinical spectrum, such as disorders of consciousness (DOC), or a particular manipulation, such as anesthesia induction. In the former case, however, the ground truth may not be fully known due to a high rate of misdiagnosis^9–11^, whereas in the latter case, generalizability to instances of cortical pathology may be limited.

Here, we introduce an alternative approach that leverages two rare genetic disorders with abnormal EEG phenotypes to challenge EEG biomarkers of conscious state by testing their generalizability across diverse oscillatory regimes. Specifically, we examined (1) a cohort of children with Angelman syndrome (AS), caused by deletions of maternal 15q11.2-q13.1 and other etiologies that inhibit *UBE3A* gene expression^12^, (2) a cohort of children with the opposite genetic lesion, duplication 15q11.2-q13.1 syndrome (Dup15q)^13^, and (3) a cohort of neurotypical (NT) children. Despite severe developmental delays in AS and Dup15q, all three groups exhibit the conventional behavioral traits associated with consciousness during wakefulness and diminished consciousness during NREM sleep. For instance, children with AS show the same level of social imitation of novel actions as NT children^14^, and children with AS who are nonverbal may communicate using gestures or alternative communication devices^15,16^. Nonetheless, the EEG phenotypes of these three groups are starkly different. Specifically, children with AS display low-frequency delta activity in their wake EEGs typical of slow-wave sleep or anesthesia, even when the children are fully awake and conscious^17^. Conversely, children with Dup15q display high-frequency beta activity in their EEGs that can persist into NREM sleep, often resulting in no identifiable slow-wave sleep^18^. Utilizing these two rare genetic disorders together with NT children, we have created a testbed for biomarkers of conscious state.

To assess whether the sensitivity of EEG entropy for consciousness is upper-bounded by that of spectral EEG features, we used binary classifiers to identify features that are sensitive to consciousness even under abnormal conditions in AS and then applied these features to two independent validation sets: wake and NREM sleep EEGs from NT children and children with Dup15q. Specifically, we examined the following EEG features: (1) absolute and relative spectral power as SC measures, and an FC measure, the debiased weighted phase lag index (dwPLI), in six octaves: slow (0.5–1.0 Hz), δ1 (1–2 Hz), δ2 (2–4 Hz), θ (4–8 Hz), α-σ (8–16 Hz), and β (16–32 Hz), and (2) signal entropy, including SC measures such as LZ and context tree weighting (CTW), modified multiscale entropy (mMSE), permutation entropy (PermEn), and an FC measure, the weighted symbolic mutual information (wSMI), which captures spatiotemporal complexity. For detailed descriptions of the EEG features, see “Methods”. Our results show that entropy measures convey information beyond that of spectral measures and outperform them as biomarkers for detecting consciousness across oscillatory regimes.

## Results

We acquired spontaneous wake and NREM sleep EEG from NT children, children with AS, and children with Dup15q. See Table 1 for details including sample size, age, sex, and data length and Supplementary Data 1 for channel-averaged EEG feature values and demographic variables. Written consent to participate in the study was obtained from families according to the Declaration of Helsinki and was approved by the institutional review boards of the participating sites. All data were collected as spontaneous 19-channel EEG using the international 10–20 montage. Data containing seizures were not analyzed. Note that no participant in our study had a disorder of consciousness, i.e., periods during which participants were awake coincided with consciousness in both NT children and children with 15q disorders.

**Table 1:** Sample size, age, sex ratio, and data length for each cohort.

Cohort	N	Age (months)	Sex ratio (M:F)	Wake data length (minutes)	Sleep data length (minutes)
AS (all EEGs)	43	50.2 ± 30.8	29:18	14.2 ± 24.3	18.7 ± 18.4
AS (participants)	34	50.6 ± 30.7	10:7	15.9 ± 27.4	19.3 ± 20.1
NT	37	79.9 ± 13.9	11:10	5.7 ± 3.4	13.1 ± 7.2
Dup15q	11	69.3 ± 38.1	6:5	18.1 ± 4.5	28.3 ± 10.0

Age and data length are reported above as mean ± std. Because some participants with Angelman syndrome (AS) had multiple EEGs, we report separate descriptives for all EEGs (first row) and for unique participants after first averaging within participants (second row). *N* = 25 participants with AS had partial deletions of 15q, with the remaining participants having other etiologies, e.g., *UBE3A* point mutations. *N* = 26 participants with AS had one dataset retained following preprocessing, *N* = 7 participants had two datasets retained, and *N* = 1 participant had three datasets retained. *N* = 9 participants with Dup15q had an extranumerary chromosome (i.e., isodicentric duplication or tetrasomy 15q) while the remaining *N* = 2 participants had interstitial duplications (i.e., trisomy 15q). See Extended Data for channel-averaged EEG feature values, as well as age, sex, genotype, cohort, conscious state, and usable data length for each participant’s dataset.

### Approach and rationale

Given that oscillatory spectral features are dissociated from conscious state in AS and Dup15q, we asked whether EEG entropy features would show a similar dissociation. We thus compared spectral and entropy EEG features head-to-head as biomarkers of consciousness to determine whether the classification performance of entropy features is upper-bounded by that of spectral EEG features. Although we previously investigated this question in AS alone^17^, our current investigation builds on the prior study by using EEG data from AS together with data from its genetic converse, Dup15q, as well as NT children, to build a testbed for finding EEG biomarkers of conscious state that generalize across diverse oscillatory regimes. In the main analysis, we chose sets of spectral and entropy features based on their ability to separate wakefulness and NREM sleep in children with AS; we then trained binary classifiers on these features in AS data and validated these classifiers using NT and Dup15q data. This approach evaluated whether features that distinguish consciousness from unconsciousness in AS generalized to other oscillatory regimes, both normal (NT) and abnormal (Dup15q). A possible shortcoming of this approach, however, is that even if the features yielded generalize to other oscillatory regimes, the feature selection is arguably idiosyncratic to AS and thus overly restrictive. For this reason, we also performed a supplementary analysis in which we chose sets of spectral and entropy features based on their ability to separate wakefulness and NREM sleep in NT children with normal EEGs. Binary classifiers were then trained using these EEG features in NT data and validated using abnormal EEGs from children with AS and Dup15q.

**Fig. 1:**
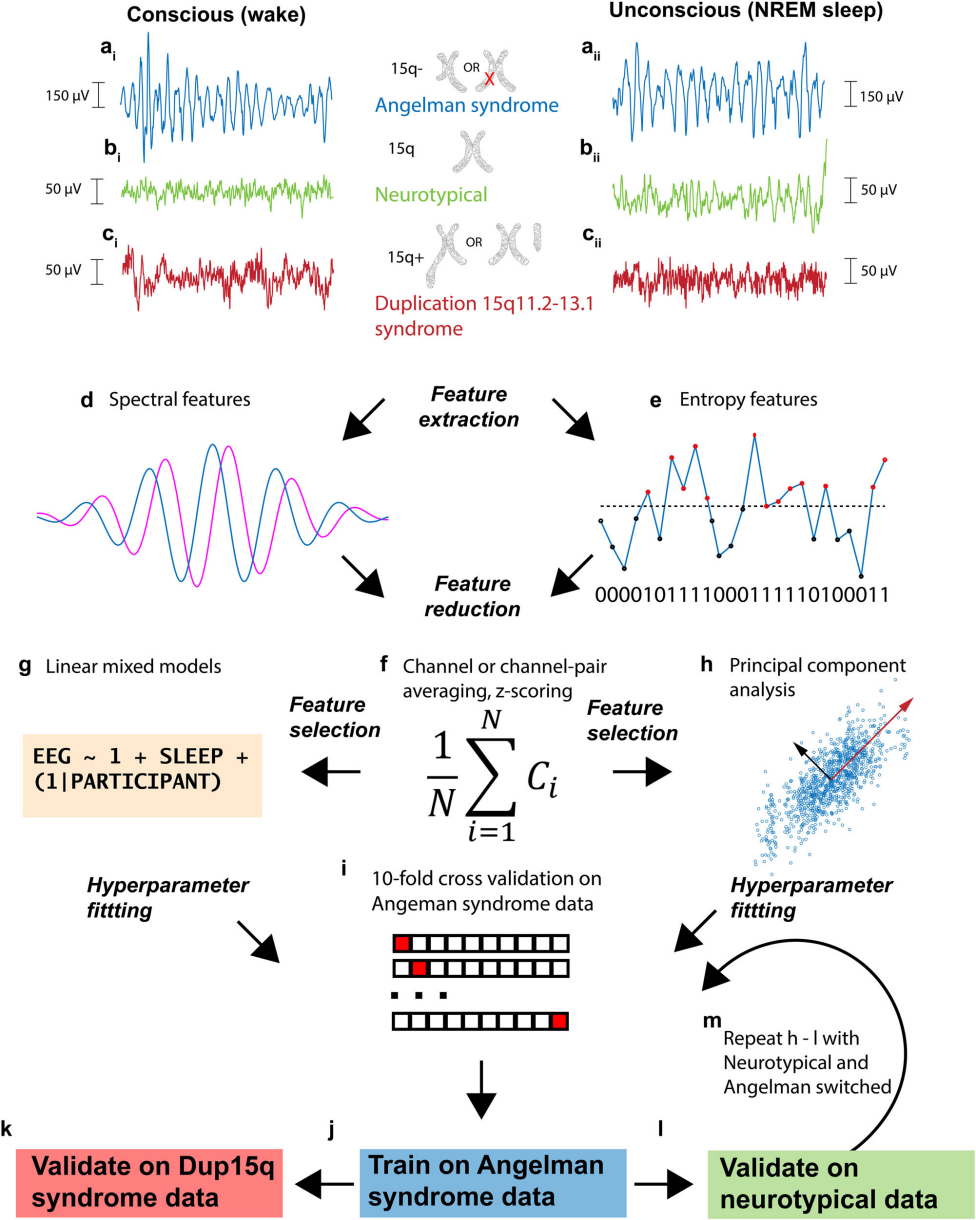
Schematic overview of the data analysis pipeline. Data from children with AS, NT children, and children with Dup15q were analyzed during wakefulness and NREM sleep. Children with AS generally have either genetic mutations or partial 15q deletions affecting the gene *UBE3A*, and an abnormal, high voltage delta EEG phenotype during both wakefulness (**a**_**i**_) and NREM sleep (**a**_**ii**_). NT children generally display low voltage, fast EEG activity during wakefulness (**b**_**i**_) and high amplitude, slow activity during NREM sleep (**b**_**ii**_). Children with Dup15q have partial trisomy or tetrasomy of 15q and an abnormal EEG phenotype characterized by fast β activity during wakefulness (**c**_**i**_) and, to some extent, NREM sleep (**c**_**ii**_). We extracted spectral (**d**) and entropy (**e**) features from wake and NREM sleep EEG recordings from the above cohorts. We then computed the mean across channels (a global average for single channel measures) or channel-pairs (a short-range or long-range average for functional connectivity measures) and subsequently z-scored these values (**f**). Next, we used two approaches for machine learning: feature selection was performed using linear mixed models (LMMs) to find features in each category that best differentiated wake from NREM sleep in AS as judged by regression coefficients (**g**). As an alternative, we also performed feature selection using PCA on the wakefulness—sleep feature contrast in AS and utilized the loadings from the first N PCs needed to explain ≥90% of the variance (**h**). In both approaches, we fit the regularized hyperparameter using 10-fold cross validation on AS data (**i**). Having determined this parameter, we then trained a regularized logistic regression classifier on AS data (**j**) and utilized two separate validations sets comprised of Dup15q (**k**) and NT (**l**) data. Finally, we repeated analysis steps **h**–**l** with the roles of NT and AS data switched (**m**), i.e., we trained classifiers on NT data and used Dup15q and AS data as validation sets. For channel-averaged EEG feature values and demographic variables, see Supplementary Data 1.

EEG features were averaged across all 19 channels for SC measures or separately across short-range and long-range channel pairings for FC measures (Supplementary Fig. 1). Following averaging, all EEG features were pooled across cohorts and z-scored. We then utilized two complementary approaches for EEG feature selection. In one approach, we fit linear mixed models (LMMs) to each feature in the AS data, using conscious state and random intercepts to predict EEG features. We then selected features with large regression coefficients for conscious state, specifically, |β| > 0.5. In another approach, we applied principal component analysis (PCA) to EEG features (wakefulness – sleep) derived from AS data to exploit interactions between features. PCA was applied separately to EEG features in each category (see Table 2 for categories). For each feature category, we then selected the minimum number of PCs needed to explain at least 90% of the variance, and PCA loadings were then applied to data from all participants. Unlike the former approach using LMMs, this approach may take advantage of the potentially synergistic interactions between individual EEG features which combine into PCs. Having selected features, regularized logistic regression (RLR) models were subsequently tuned using cross-validation for hyperparameter selection, trained within the AS data, and evaluated on two external datasets (NT and Dup15q) to assess classification performance. See Fig. 1 for an overview of the data analysis pipeline and Table 2 for a complete list of EEG feature abbreviations.

**Table 2:** Abbreviations for EEG features.

Abbreviation	EEG feature name	EEG feature type	Betas	Selected
mMSE	Modified multiscale entropy	scEntropy	−0.43	FALSE
LZ	Lempel-Ziv complexity	scEntropy	−0.42	FALSE
CTW	Context-tree weighted complexity	scEntropy	−0.44	FALSE
PermEn8	16–40 Hz Permutation entropy (τ = 8 ms)	scEntropy	−1.09	TRUE
PermEn16	8–20 Hz Permutation entropy (τ = 16 ms)	scEntropy	−0.68	TRUE
PermEn32	4–10 Hz Permutation entropy (τ = 32 ms)	scEntropy	−0.57	TRUE
PermEn64	2–5 Hz Permutation entropy (τ = 64 ms)	scEntropy	−1.56	TRUE
PermEn128	1–2.5 Hz Permutation entropy (τ = 128 ms)	scEntropy	0.03	FALSE
SRwSMI8	Short-range 16–40 Hz weighted symbolic mutual information (τ = 8 ms)	fcEntropy	1.12	TRUE
SRwSMI16	Short-range 8–20 Hz weighted symbolic mutual information (τ = 16 ms)	fcEntropy	0.24	FALSE
SRwSMI32	Short-range 4–10 Hz weighted symbolic mutual information (τ = 32 ms)	fcEntropy	−0.22	FALSE
SRwSMI64	Short-range 2–5 Hz weighted symbolic mutual information (τ = 64 ms)	fcEntropy	0.16	FALSE
SRwSMI128	Short-range 1–2.5 Hz weighted symbolic mutual information (τ = 128 ms)	fcEntropy	0.21	FALSE
LRwSMI8	Long-range 16–40 Hz weighted symbolic mutual information (τ = 8 ms)	fcEntropy	−0.69	TRUE
LRwSMI16	Long-range 8–20 Hz weighted symbolic mutual information (τ = 16 ms)	fcEntropy	−0.56	TRUE
LRwSMI32	Long-range 4–10 Hz weighted symbolic mutual information (τ = 32 ms)	fcEntropy	−0.60	TRUE
LRwSMI64	Long-range 2–5 Hz weighted symbolic mutual information (τ = 64 ms)	fcEntropy	−0.57	TRUE
LRwSMI128	Long-range 1–2.5 Hz weighted symbolic mutual information (τ = 128 ms)	fcEntropy	0.03	FALSE
sA	Absolute 0.5–1.0 Hz (slow) power	scSpectralA	0.00	FALSE
δ1A	Absolute 1–2 Hz (delta1) power	scSpectralA	0.92	TRUE
δ2A	Absolute 2–4 Hz (delta2) power	scSpectralA	0.35	FALSE
θA	Absolute 4–8 Hz (theta) power	scSpectralA	0.16	FALSE
α-σA	Absolute 8–16 Hz (alpha-sigma) power	scSpectralA	0.50	TRUE
βA	Absolute 16–32 Hz (beta) power	scSpectralA	−0.04	FALSE
sR	Relative 0.5–1.0 Hz (slow) power	scSpectralR	0.03	FALSE
δ1R	Relative 1–2 Hz (delta1) power	scSpectralR	1.10	TRUE
δ2R	Relative 2–4 Hz (delta2) power	scSpectralR	−0.18	FALSE
θR	Relative 4–8 Hz (theta) power	scSpectralR	−0.85	TRUE
α-σR	Relative 8–16 Hz (alpha-sigma) power	scSpectralR	−0.03	FALSE
βR	Relative 16–32 Hz (beta) power	scSpectralR	−0.10	FALSE
SRdwPLIs	Short-range 0.5–1.0 Hz (slow) debiased weighted phase lag index	fcSpectral	0.03	FALSE
SRdwPLIδ1	Short-range 1–2 Hz debiased weighted phase lag index	fcSpectral	0.25	FALSE
SRdwPLIδ2	Short-range 2–4 Hz debiased weighted phase lag index	fcSpectral	−0.01	FALSE
SRdwPLIθ	Short-range 4–8 Hz (theta) debiased weighted phase lag index	fcSpectral	−0.51	TRUE
SRdwPLIα-σ	Short-range 8–16 Hz (alpha-sigma) debiased weighted phase lag index	fcSpectral	0.21	FALSE
SRdwPLIβ	Short-range 16–32 Hz (beta) debiased weighted phase lag index	fcSpectral	0.32	FALSE
LRdwPLIs	Long-rage 0.5–1.0 Hz (slow) debiased weighted phase lag index	fcSpectral	0.00	FALSE
LRdwPLIδ1	Long-rage 1–2 Hz debiased weighted phase lag index	fcSpectral	0.22	FALSE
LRdwPLIδ2	Long-rage 2–4 Hz debiased weighted phase lag index	fcSpectral	−0.02	FALSE
LRdwPLIθ	Long-rage 4–8 Hz (theta) debiased weighted phase lag index	fcSpectral	−0.54	TRUE
LRdwPLIα-σ	Long-rage 8–16 Hz (alpha-sigma) debiased weighted phase lag index	fcSpectral	0.21	FALSE
LRdwPLIβ	Long-rage 16–32 Hz (beta) debiased weighted phase lag index	fcSpectral	0.18	FALSE

The column “Betas” gives the beta coefficients from the term CONSCIOUS in the linear mixed model (LMM) used to predict the EEG feature after training on AS data. The column “Selected” indicates whether each feature was selected for machine learning classification based on |β| > 0.5.

*scEntropy* single-channel entropy, *fcEntropy* entropy-based functional connectivity, *scSpectralA* absolute spectral power, *scSpectralR* relative spectral power, *fcSpectral* spectral-based functional connectivity.

### Behavioral responsiveness

Developmental assessments, performed for nearly all children with AS (97% of participants) and Dup15q (91% of participants), quantified each child’s behavioral responsiveness during wakefulness, e.g., in the form of receptive and expressive language abilities (see “Methods”). Note that these assessments were not performed during EEG, as this would have been impractical given delayed developmental abilities in these children. However, in the case of children with AS, assessments were usually performed the same day as EEG (65% of recordings) or within one day of EEG (84% of recordings). During wakefulness, children with AS and Dup15q were behaviorally responsive and clearly conscious, as indicated by measurable expressive and receptive language abilities (see Supplementary Table 1 and Supplementary Table 2). Note that this is consistent with results of both the AS Natural History Study (*n* = 250)^19^ and a prior study of Dup15q (*n* = 41)^20^ in which all children displayed measurable communication abilities, even if they were considered “nonverbal” in the conventional sense. If, conversely, it were the case that some children lacked behavioral responsiveness, one would expect to see a flooring effect in these data. In fact, no flooring effect was observed in our data or the studies cited above.

### EEG spectral power and connectivity

**Fig. 2:**
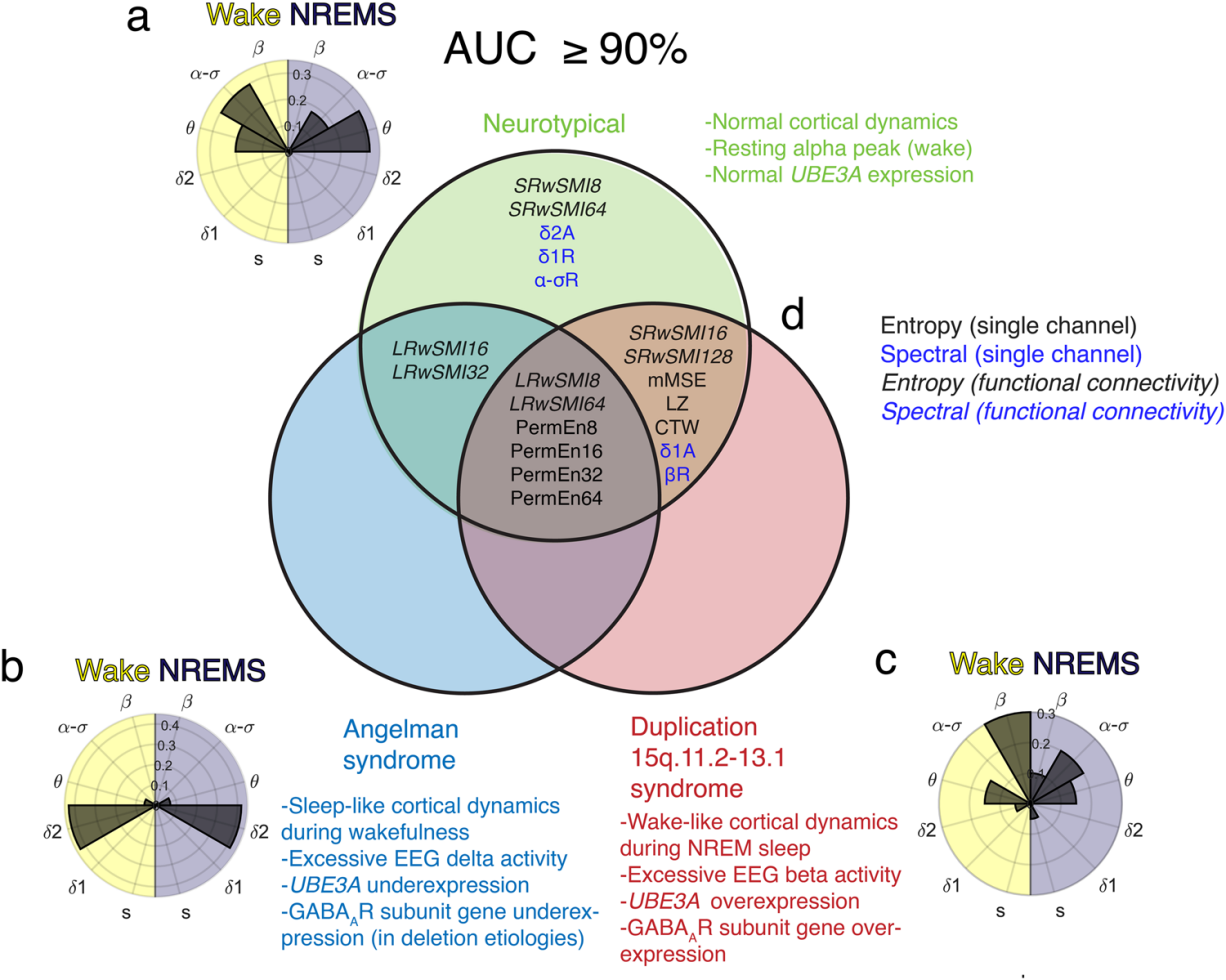
EEG entropy measures of neural complexity are the common denominator of consciousness under conditions of both normal (neurotypical) and abnormal (Angelman syndrome, Duplication 15q11.2-13.1 syndrome) cortical dynamics. The above histograms report the modal peak frequency across channels for NT children (**a**), children with AS (**b**), and children with Dup15q (**c**). The Venn diagram reports EEG features that yield an area under the receiver operating characteristics curve (AUC) ≥ 90% for NT children (green circle, **a**), children AS (blue circle, **b**), and children with Dup15q (red circle, **c**). Abbreviations of entropy features are written in black font, spectral features in blue font, and functional connectivity (FC) features in italicized font. The intersection of all three groups is reported in (**d**) and contains only entropy features, thus demonstrating that entropy is a common denominator of consciousness across different populations with diverse cortical dynamics. Source data are presented in Supplementary Data 2 (a, b, c) and Supplementary Data 3 (d). NREMS Non-rapid eye movement sleep.

Most NT participants (*N* = 37) exhibited an α-σ band EEG peak during wakefulness, with a slowing toward the θ band as the modal peak frequency across channels during NREM sleep (Fig. 2a). By contrast, nearly all EEGs from participants with AS (*N* = 34 participants, 43 EEGs) showed peaks in the δ2 band during both wakefulness and NREM sleep (Fig. 2b). Also, note that even beyond the δ2 band, absolute power for participants with AS during wakefulness is greater than that of the other two cohorts at nearly all frequencies lower than the β band (Supplementary Fig. 2). The modal EEG peak frequency of participants with Dup15q (*N* = 11) appeared in the β band during wakefulness, with a slowing toward the α-σ band during NREM sleep (Fig. 2c). The Dup15q cohort also showed substantially greater variance in EEG power than other cohorts (Supplementary Fig. 2c, d) as indicated by 95% confidence intervals (CIs). Note that no peak was detected for two participants with Dup15q during wakefulness and one participant with Dup15q during NREM sleep. See Supplementary Fig. 2 for channel-averaged spectra for each group during wakefulness and NREM sleep and Supplementary Data 2 for EEG peak data.

For all three cohorts, both short-range and long-range dwPLI spectra appeared highly similar (Supplementary Fig. 3). Participants with AS displayed the greatest dwPLI during wakefulness in the δ2 and θ bands; during sleep, dwPLI is was elevated in the δ2 band, but somewhat diminished in the θ band (Supplementary Fig. 3a, b). As with spectral power, dwPLI also appeared highly similar between wakefulness and sleep in AS, with the greatest connectivity occurring in the δ2 and θ bands (Supplementary Fig. 3a, b), whereas NT and Dup15q cohorts generally exhibited greater dwPLI values during sleep. Participants with Dup15q showed elevated dwPLI in the slow (s) and low α-σ bands during sleep but not wakefulness (Supplementary Fig. 3c, d). Finally, NT participants (Supplementary Fig. 3e, f) showed generally elevated dwPLI during sleep, with large peaks in the θ and α-σ bands. The maximum dwPLI in the α-σ band occurred at a lower frequency during wake than during sleep.

### Machine learning classification

**Fig. 3:**
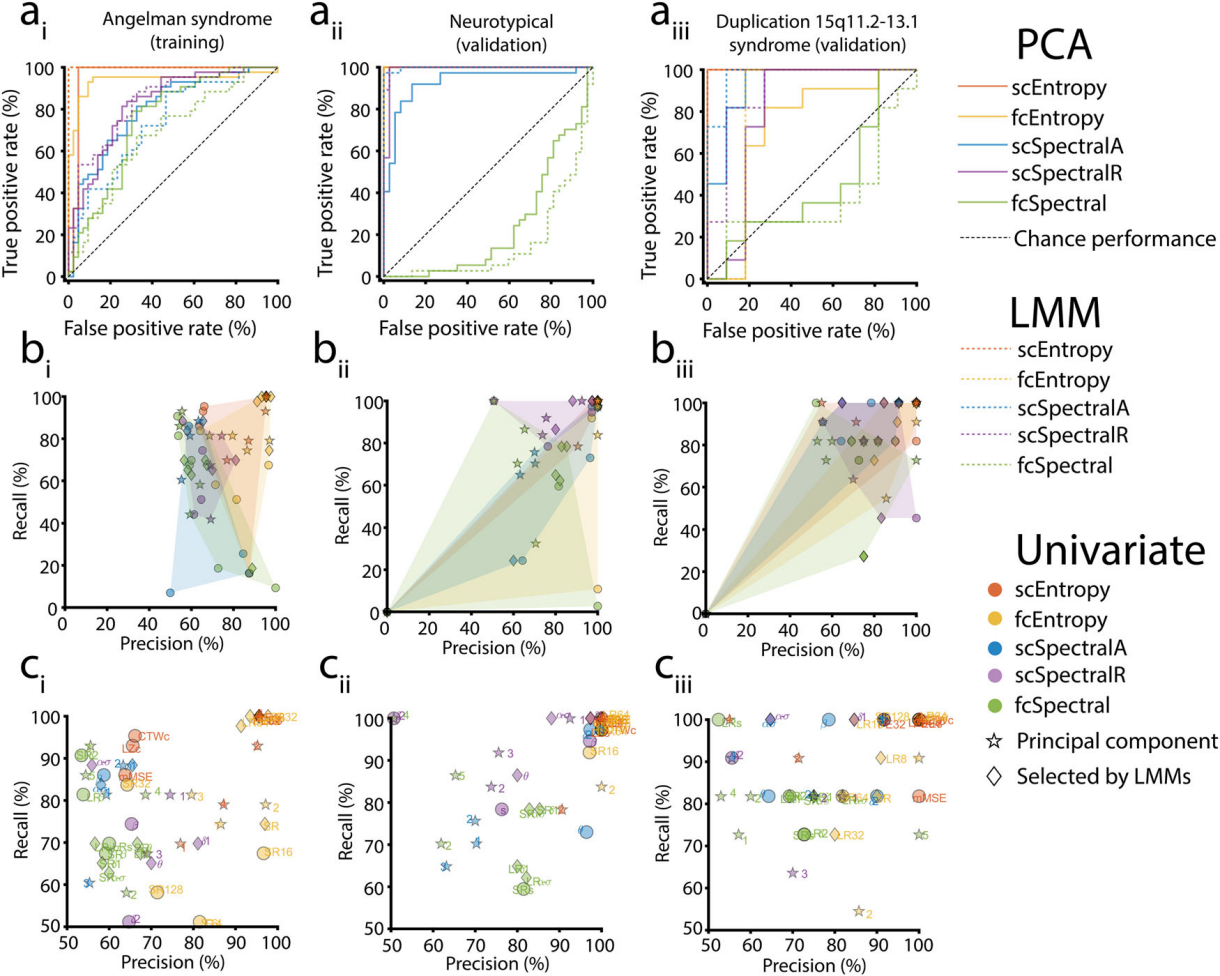
Classification performance of EEG features in each cohort. Receiver operating characteristic curves color-coded by category (**a**) for each the AS training set (1), NT validation set (2), and the Dup15q validation set (3) all show larger AUCs for entropy features than for corresponding (i.e., single-channel or functional connectivity) spectral features. Features selected using PCA are shown with solid lines and features selected using LMMs are shown with dashes lines. To visualize the usefulness of models trained on individual features, we plotted precision (i.e., positive predictive value) versus recall (i.e., sensitivity) for each feature color-coded by category (**b**); regularization was not performed for univariate models. Diamonds represent features selected using LMMs and stars represent PCs of a given feature category. To better visualize individual features, we separately plotted the upper quadrant of each precision versus recall plot with features labeled (**c**). For brevity, abbreviations are shorted to cut information given in color-coded labels, i.e., yellow symbols omit “wSMI”, green symbols omit “dwPLI”, blue symbols omit “A”, and purple symbols omit “R”. PCs (stars) are labeled according to rank (proportion of variance explained). Source data are presented in Supplementary Data 4 (a) and Supplementary Data 3 (b, c).

**Fig. 4:**
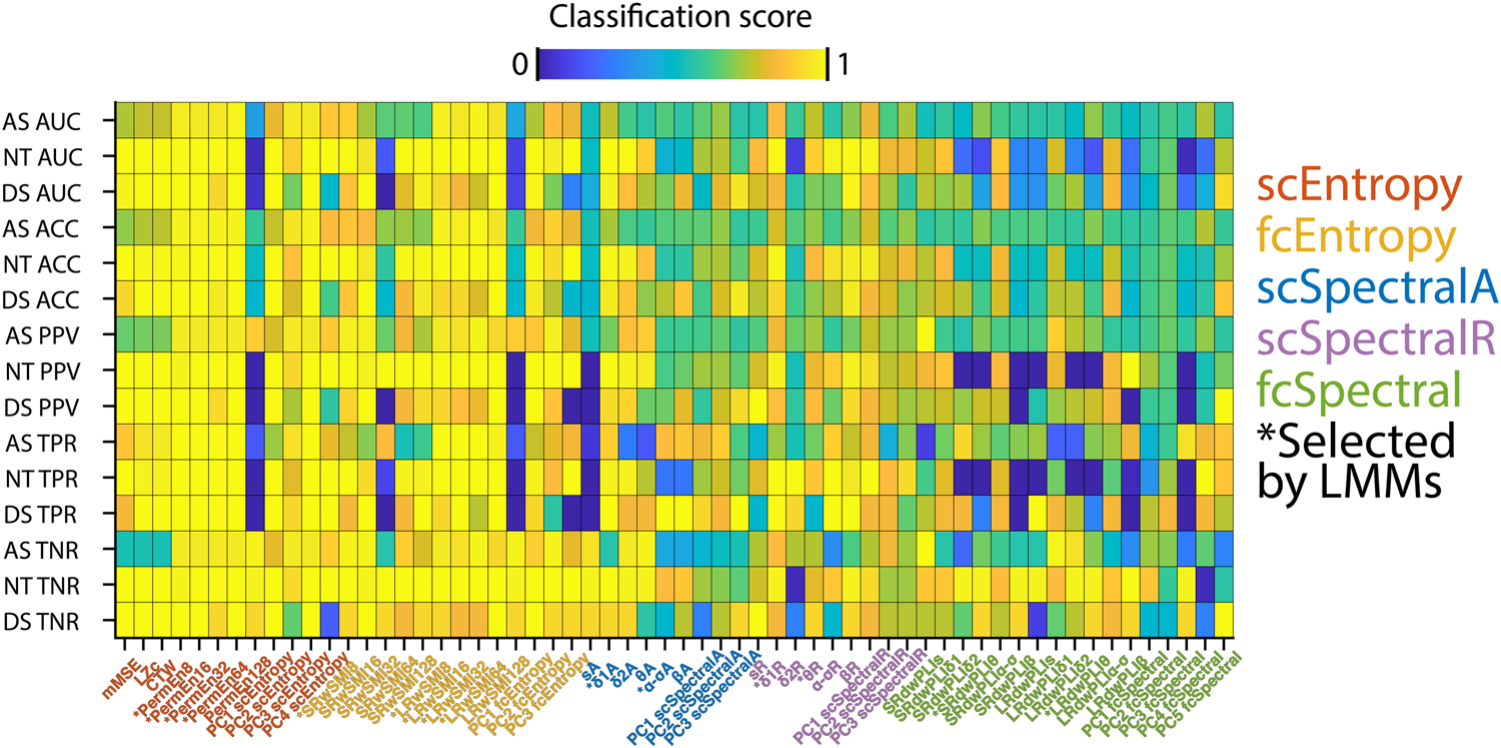
Unregularized logistic regression univariate classifier performance for all channel-averaged variables. Model fitting was performed without regularization for univariate classification. AUC area under ROC curve, ACC accuracy, PPV positive predictive value (precision), TPR true positive rate (recall or sensitivity), TNR true negative rate (specificity). Asterisks denote features selected using LMMs. Source data are presented in Supplementary Data 3.

Using simulated data, we confirmed that our choice of window length for entropy measures was appropriate (Supplementary Fig. 4). Moreover, in our actual data, we confirmed using LMMs that EEG data length did not significantly influence EEG feature estimates (Supplementary Fig. 5); see Supplementary Results for further details. In RLR models fitted prior to averaging features across channels, we observed low spatial variability of classifier performance in scalp topographies (Supplementary Fig. 6, Supplementary Fig. 7), with the only exception being three frontal channels (F7, F8, and Fp1) in the NT validation set (Supplementary Fig. 7aii). This local noise, possibly related to ocular artifacts which could reduce signal entropy during wakefulness, washes out in the spatial average we used to evaluate classifiers, thus supporting our decision to average features across channels. To inspect the performance of each individual feature, we performed univariate classifications without regularization and computed the area under the curve (AUC), accuracy, precision, recall, and specificity for all features (Figs. 3, 4; see Supplementary Data 3). The results show an overall pattern of stronger performance for entropy rather than spectral features (Fig. 2d, Supplementary Fig. 8). For the results that follow, we selected between 2 and 5 individual EEG features in each category listed in Table 2 based on LMMs and 2–5 PCs which explained at least 90% of the wakefulness—NREM sleep variance in AS data in each category (see Table 2 for selected features and beta coefficients from LMMs). For PCA loadings and normalized eigenvalues, as well as regression coefficients from LMMs, see Supplementary Fig. 9 and Supplementary Fig. 10. For receiver operating characteristic (ROC) curve data, see Supplementary Data 4.

**Fig. 5:**
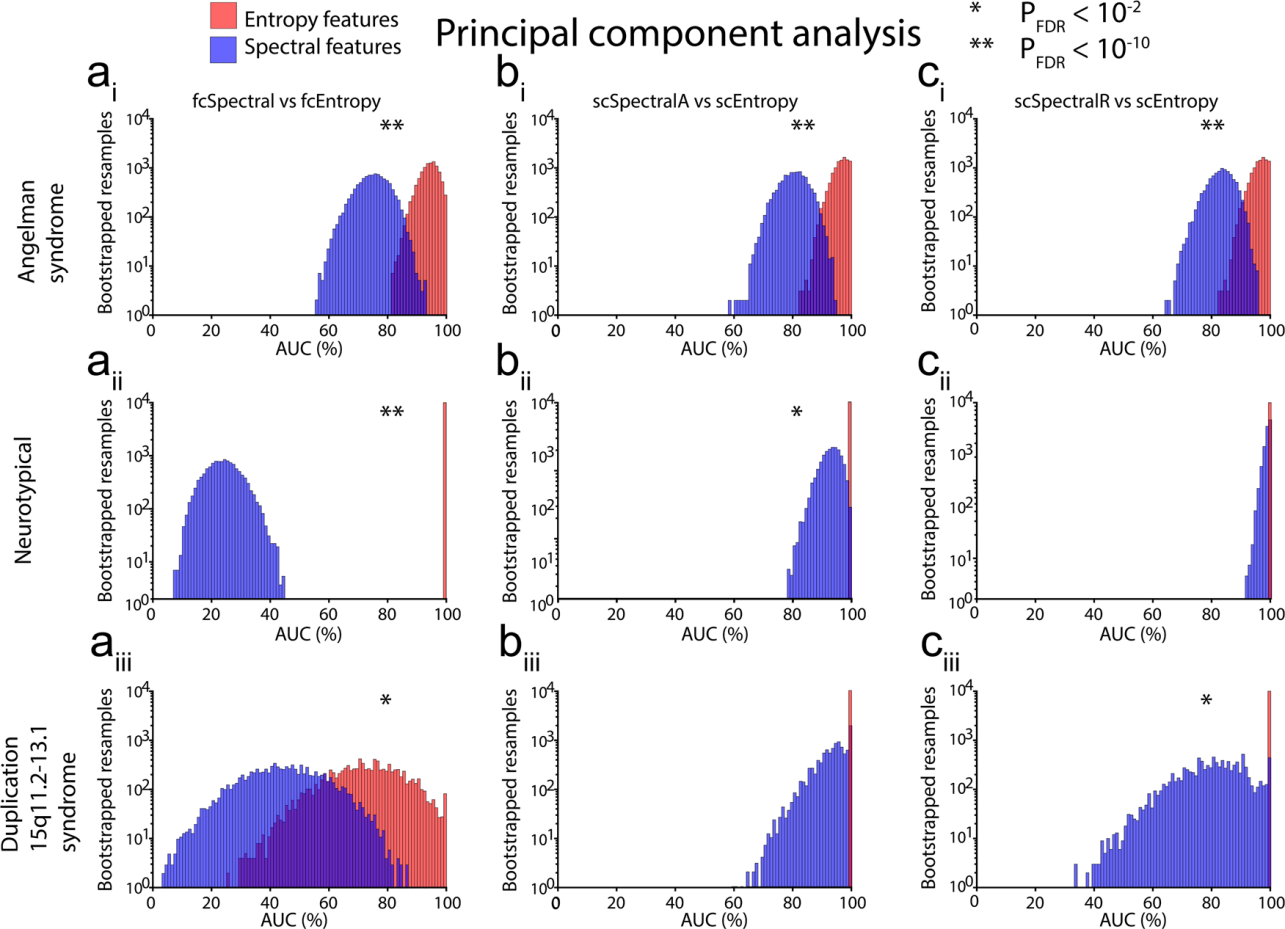
Comparison of areas under the receiver operating characteristics curve (AUCs) for entropy (red) and spectral (blue) features selected using principal component analysis (PCA). We performed three basic comparisons (**a** fcEntropy vs fcSpectral; **b** scEntropy vs scSpectralA; **c** scEntropy vs scSpectralR) with EEG data from three datasets [AS (top row) training data; NT (middle row) validation data; Dup15q (bottom row) validation data]. In each case, we generated 10000 bootstrapped resamples to derive AUC confidence intervals in Supplementary Table 3. Note that the number of resamples on the vertical axis of each histogram is log-scaled due to the very large range of resamples across histogram bins. Also note that classifier performance was saturated using entropy features for all comparisons using NT data and two comparisons using Dup15q data, i.e., all bootstrapped resamples yielded 100% AUC for entropy features in these instances. Seven out of the nine comparisons above yield significantly larger AUCs for entropy features, as indicated by asterisks. Source data are presented in Supplementary Data 5.

**Fig. 6:**
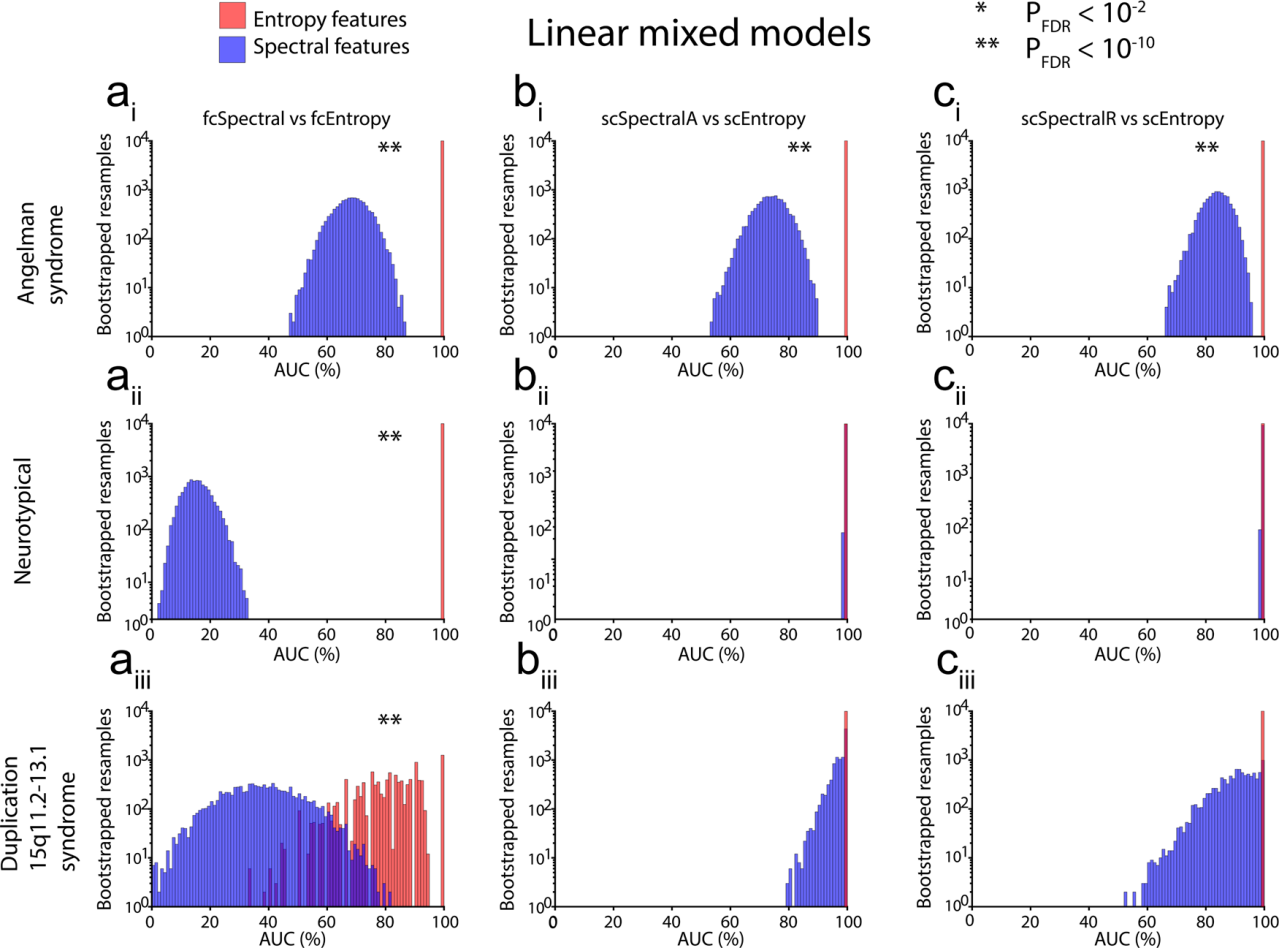
Comparison of areas under the receiver operating characteristics curve (AUCs) for entropy (red) and spectral (blue) features selected using linear mixed models (LMMs). As in Fig. 5, we performed three basic comparisons (**a** fcEntropy vs fcSpectral; **b** scEntropy vs scSpectralA; **c** scEntropy vs scSpectralR) with EEG data from three datasets [AS (top row) training data; NT (middle row) validation data; Dup15q (bottom row) validation data]. In each case, we generated 10,000 bootstrapped resamples to derive AUC confidence intervals in Supplementary Table 3. Note that the number of resamples on the vertical axis of each histogram is log-scaled due to the very large range of resamples across histogram bins. Also note that classifier performance was saturated using entropy features for all comparisons except for a_iii_ (Dup15q FC features), i.e., all bootstrapped resamples yielded 100% AUC for entropy features in these instances. Five out of the nine comparisons above yield significantly larger AUCs for entropy features, as indicated by asterisks. Source data are presented in Supplementary Data 5.

For 12 out of 18 comparisons of entropy versus spectral features listed in Table 3, entropy features performed significantly better than spectral features [Figs. 5, 6, *p* < 0.05, false discovery rate (FDR) corrected], and in nine such comparisons, results were highly significant (Figs. 5, 6, *p* < 10^−10^, FDR corrected); see Supplementary Data 5 for AUC histogram bin data (bootstrapped resamples). In those comparisons for which entropy features did not perform significantly better than spectral features, we noted performance saturation, i.e., RLR classifiers yielded AUCs > 90% for both entropy and spectral features. Spectral features never yielded larger AUCs than entropy features, i.e., AUCs were numerically larger for entropy features than for spectral features in all 18 comparisons, even those that were not significant. Most classifiers (25 out of 30) performed significantly better than chance (Supplementary Table 3, *p* < 0.05 using one-tailed tests, FDR corrected). Of the remaining five classifiers, four were trained on fcSpectral features and yielded AUCs < 50%, i.e., these classifiers yielded worse-than-chance performance, though our one-tailed tests only evaluated statistical significance for better-than-chance performance. Similar results were obtained even when the AUC for AS was computed with 10-fold cross-validation rather than from training performance (Supplementary Table 4). To test the robustness of k in the k-fold cross-validation used to fit the regularization parameter and to report cross-validation performance in AS, we also performed the analysis with 5-fold cross-validation and observed highly similar results (Supplementary Table 5). A supplementary analysis also directly compared entropy and spectral EEG features after performing feature selection and training based on NT data, with results that were also favorable to entropy features (see Supplementary Results, Tables S6 and S7).

**Table 3:** Comparison of areas under the curve (AUC) of receiver operating characteristic plots for entropy versus spectral measures.

Train on	Feature selection	Cohort	*N*	Test	Larger AUC	P_FDR_	Entropy AUC	Spectral AUC
AS	PCA	AS	43	fcEntropy vs fcSpectral	Entropy	1.40E−41	0.938	0.747
AS	PCA	NT	37	fcEntropy vs fcSpectral	Entropy	2.40E−119	1.000	0.240
AS	PCA	Dup15q	11	fcEntropy vs fcSpectral	Entropy	2.70E−05	0.719	0.446
AS	PCA	AS	43	scEntropy vs scSpectralA	Entropy	4.75E−29	0.962	0.803
AS	PCA	NT	37	scEntropy vs scSpectralA	Entropy	2.40E−05	1.000	0.928
AS	PCA	Dup15q	11	scEntropy vs scSpectralA	Entropy	0.352	1.000	0.934
AS	PCA	AS	43	scEntropy vs scSpectralR	Entropy	1.11E−19	0.962	0.832
AS	PCA	NT	37	scEntropy vs scSpectralR	Entropy	0.557	1.000	0.988
AS	PCA	Dup15q	11	scEntropy vs scSpectralR	Entropy	0.002	1.000	0.802
AS	LMM	AS	43	fcEntropy vs fcSpectral	Entropy	3.16E−112	1.000	0.683
AS	LMM	NT	37	fcEntropy vs fcSpectral	Entropy	2.40E−119	1.000	0.156
AS	LMM	Dup15q	11	fcEntropy vs fcSpectral	Entropy	8.14E−12	0.818	0.380
AS	LMM	AS	43	scEntropy vs scSpectralA	Entropy	6.54E−77	1.000	0.738
AS	LMM	NT	37	scEntropy vs scSpectralA	Entropy	0.932	1.000	0.998
AS	LMM	Dup15q	11	scEntropy vs scSpectralA	Entropy	0.774	1.000	0.975
AS	LMM	AS	43	scEntropy vs scSpectralR	Entropy	6.19E−31	1.000	0.836
AS	LMM	NT	37	scEntropy vs scSpectralR	Entropy	0.916	1.000	0.997
AS	LMM	Dup15q	11	scEntropy vs scSpectralR	Entropy	0.142	1.000	0.901

All *P*-values are FDR corrected (P_FDR_) for multiple testing.

*AS* Angelman syndrome, *NT* neurotypical, *Dup15q* duplication 15q11.2-q13.1 syndrome, *PCA* principal components analysis, *LMM* linear mixed model, *scEntropy* single-channel entropy, *fcEntropy* entropy-based functional connectivity, *scSpectralA* absolute spectral power, *scSpectralR* relative spectral power, *fcSpectral* spectral-based functional connectivity.

Univariate classifications without regularization demonstrated that only entropy features achieved AUCs ≥ 90% for all three datasets (Fig. 2d, Fig. 4; see Supplementary Data 3). Specifically, the features that achieved this high level of performance were PermEn8, PermEn16, PermEn32, PermEn64, LRwSMI8, and LRwSMI64. No spectral EEG features achieved the same level of univariate classification performance across all three datasets. When using other performance measures, such as accuracy, precision (i.e., positive predictive value), and sensitivity (i.e., true positive rate or recall), we also found that only entropy features achieved ≥ 90% performance across all three datasets (Supplementary Fig. 8a–c, Fig. 3b, c). Only specificity [i.e., true negative rate, or 1 - (false positive rate)] was an exception in this regard, with spectral features sA and δ2A achieving ≥ 90% specificity in all three datasets (Supplementary Fig. 8d). These spectral features rarely mislabeled NREM sleep as consciousness (likely because low frequency oscillations indicating unconsciousness are present in the AS dataset during NREM sleep) but more often failed to detect consciousness (likely because low frequency oscillations are also present in the AS dataset during wakefulness).

### Entropy decomposition

**Fig. 7:**
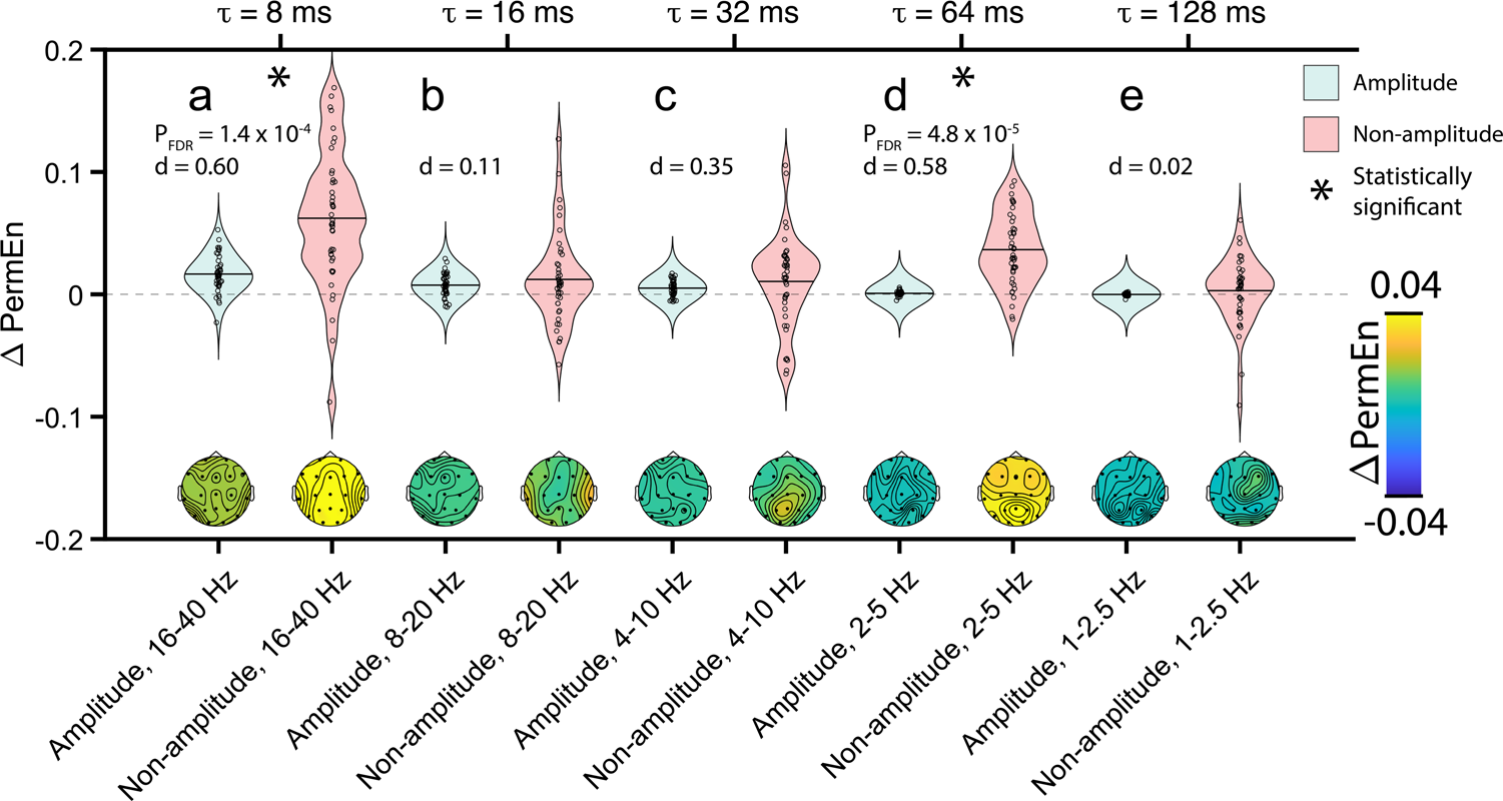
Permutation Entropy (PermEn) decomposition. Violin plots (top row) display ΔPermEn in AS between wakefulness and NREM sleep driven by amplitude (blue-gray) and non-ampltidue amplitude (pink) effects for **a** PermEn8, **b** PermEn16, **c** PermEn32, **d** PermEn64, and **e** PermEn128. Positive values of ΔPermEn indicate greater PermEn during wakefulness. Solid black horizontal lines inside violin plots represent the mean of each distribution, and statistically significant comparisons of amplitude and non-amplitude contributions are marked with asterisks. Topographic scalp plots (bottom row) show the spatial distribution of ΔPermEn averaged across all participants with AS. Source data are presented in Supplementary Data 6.

To further investigate how signal entropy varies with conscious state in AS despite the presence of high amplitude delta oscillations during both NREM sleep and wakefulness, we performed an entropy decomposition that quantified the extent to which signal amplitude versus non-amplitude factors (e.g., phase or phase × amplitude interactions) drive changes in signal entropy between wakefulness and NREM sleep (see Supplementary Data 6). We focused our entropy decomposition on PermEn, given that we found large sleep/wakefulness regression coefficients in AS using PermEn measures with LMMs. As seen in Fig. 7, PermEn increased in wakefulness relative to NREM sleep in most cases. Given that we were primarily interested in whether entropy changes are due to signal amplitude, we summed together phase and interaction terms to create a non-amplitude term. For each AS dataset, we randomly sampled 20 5-s segments of usable EEG data. Below, we report statistics using FDR corrected P-values and the Cohen’s d effect sizes. Changes in PermEn due to non-amplitude factors (Fig. 7) were significantly greater than those due to signal amplitude with moderate effect sizes for PermEn8 (*p* = 0.00014, FDR corrected, *d* = 0.60) and PermEn64 (*p* = 4.83 × 10^−5^, FDR corrected, *d* = 0.58). Note that for the 1–2.5 Hz and 2–5 Hz PermEn, the entropy change driven by amplitude is very small with very low variance (Fig. 7), likely due to the AS delta EEG phenotype that is largely unchanged by conscious state. The above results demonstrate that changes in entropy that occur with NREM sleep in AS are not merely attributable to more trivial concurrent changes in signal amplitude or spectral power.

## Discussion

In this work, we obtained dissociations between cortical oscillations and consciousness in rare genetic disorders and used these disorders as a testbed for identifying EEG biomarkers of consciousness that generalize across different oscillatory regimes, including highly abnormal cortical dynamics. We found that (1) the performance of entropy or “complexity” features is not upper-bounded by the performance of spectral features, (2) entropy features generalized significantly better than spectral features for most comparisons and (3) spectral features never outperformed entropy features when classifiers were trained on AS data. Additional analyses revealed that spatial distributions of classifiers’ performances across the scalp were nearly homogeneous (Supplementary Figs. 6, 7), and that the strongest individual features were those belonging to PermEn or LRwSMI categories (Figs. 2, 3, and 4). Our results demonstrate that EEG entropy measures are highly adaptable and may perform accurately even under pathological and abnormal cortical conditions. These findings challenge current opinion in the field, as reflected by a recent survey of consciousness researchers^21^; fewer than 10% of participants listed non-integration entropy measures in their top three most trusted neural measures of consciousness, with several spectral measures, including local synchrony, ranked above entropy. In contradiction to these widely held opinions, we conclude based on our findings that consciousness largely reveals itself in bits (i.e., entropy features) rather than decibels (i.e., spectral features). Based on the generalizability of entropy features across oscillatory regimes, we conclude that entropy is a common denominator of consciousness across diverse and divergent regimes of cortical dynamics

### Beyond spectral EEG measures

Loss of consciousness often reveals itself in the form of slow spectral features such as high amplitude delta oscillations (HADOs), e.g., as observed during slow wave sleep, anesthesia, absence seizures, and DOC^4,22,23^. Nonetheless, convergent evidence from a variety of contexts suggests that HADOs are insufficient to demonstrate an absence of consciousness^1^. Individuals with AS^17,24^, Rett syndrome^25^, Lennox-Gastaut syndrome^26^, non-convulsive status epilepticus^27^, mitochondrial diseases^28^, hepatic encephalopathy^29^, post-operative delirium^30^, and “phantom” absence seizures not affecting consciousness^31^ have all demonstrated HADOs during the awake and conscious state. Similarly, pharmacological challenge with drugs such as gamma-hydroxybutyrate (GHB)^32,33^, baclofen^34^, atropine^35,36^, carbamazepine and tiagabine^37^, as well as some highly potent psychedelic tryptamines^38,39^, all induce slow EEG activity suggestive of loss of consciousness while nonetheless preserving, or even phenomenologically enhancing^38^, consciousness. Considering these lines of evidence, alternative markers of consciousness are greatly needed to detect covert consciousness amongst a background of diffuse delta waves.

Compared with spectral features, we found that entropy features that were matched on measure type (i.e., SC or FC) yielded larger AUCs under all circumstances after training models on AS data. These differences were statistically significant for the majority of comparisons and are in agreement with a recent study of neonates that found significantly greater accuracy for sleep stage classification using entropy features rather than spectral features^40^. In our study, when models were trained on NT data, the advantage afforded by entropy features appeared slightly weaker, given that the EEGs in the training set displayed normal oscillations that were useful for classification; yet, AUCs for spectral features only numerically exceeded those of entropy features in one non-significant comparison (Table 3), and AUCs were significantly greater for entropy features than spectral features in all comparisons involving AS, whose data were now used as a validation set. As revealed by an entropy decomposition, decreases in EEG entropy in AS during NREM sleep in two frequency bands (2–5 Hz and 16–40 Hz) are not driven by the signal amplitude, but rather by changes in signal phase and/or phase × amplitude interactions (Fig. 7), thus demonstrating that entropy changes with conscious state are independent of power spectral changes.

### Implications for the biology of consciousness

Several frameworks for understanding consciousness—namely, integrated information theory (IIT)^41^ and the entropic brain hypothesis (EBH)^42^—emphasize the role of complexity/entropy in consciousness. This theoretical work is bolstered by findings of decreased neural entropy during unconscious states and increased neural entropy during conscious wakefulness and, even more so, psychedelic states^5,7,38,43–47^. In particular, studies in DOC patients have highlighted PermEn and wSMI as useful entropy features for discerning the level of consciousness in patients^8,48,49^. A number of studies have also examined PermEn as an accurate marker of loss of consciousness under anesthesia^50–53^ and, more recently, sleep^54^. Building on the foregoing work, we tested whether PermEn and wSMI, among other entropy measures, would also perform well in both healthy and abnormal regimes of cortical dynamics. We found that PermEn and wSMI performed exceptionally well for all timescales except for τ = 128 ms (1– 2.5 Hz, as also found by Bourdillon et al., 2020; see Figs. 3, 4). Our study thus adds to the existing literature by confirming that PermEn and wSMI accurately detect the presence/absence of consciousness when it vanishes during NREM sleep, even in individuals with persistent HADOs (AS) or beta activity (Dup15q). More generally, our finding of larger AUCs using entropy versus spectral features supports theories (e.g., IIT and EBH) that emphasize the role of information in consciousness. Contrary to theories that emphasize cortical oscillations in consciousness^55–57^, our findings suggest that spectral phenomena are of secondary importance to consciousness versus more fundamental, complexity-based quantities. Indeed, prior work shows that spectral features^58^ are sometimes inadequate for discerning consciousness from unconsciousness^59–62^.

Given that spectral EEG features such as delta power often reflect periodic cortical down states^63^, in which pyramidal cells are hyperpolarization in a manner that likely limits consciousness^64^, why are spectral measures outperformed by entropy measures? In addition to a theoretical and fundamental identity linking certain forms of entropy/information to consciousness^65^, entropy features are also preferable over spectral features for detecting consciousness because the latter suffer from several shortcomings. Most relevant to our current study, when HADOs are used to infer unconsciousness, one must be cautious of focal generators of delta activity. These focal sources, which might be present in children with AS, appear global at the scalp level due to volume conduction, leading to a false impression of global cortical down states^17^. In addition, the overall oscillatory amplitude might be influenced by synaptic properties that do not affect consciousness, e.g., in disorders such as AS with abnormal dendritic spine density^66^. Finally, some delta oscillations may reflect hyperpolarization limited to primary sensory cortices, e.g., during states of unresponsive consciousness^67,68^. To better understand why entropy measures outperform spectral measures as biomarkers of consciousness, we direct the interested reader to Frohlich et al.^1^.

### 15q disorders as models for abnormal cortical dynamics

Our work utilizes EEG from children with AS and Dup15q to challenge and discover EEG biomarkers of conscious state with applications outside of these disorders. AS is rooted in underexpression of the gene *UBE3A*, most commonly caused by 15q11.2-q13.1 deletion^12^, and children with AS present a paradoxical EEG that strongly resembles slow wave sleep during wakefulness^1,17^. Dup15q, on the other hand, is the genetic converse of AS featuring duplication, rather than deletion, of 15q11.2-q13.1 and is characterized by an abnormally fast, rather than slow, EEG phenotype^13,69^. Given that 60% of children with Dup15q completely lack N3 slow wave sleep in overnight EEGs, there is some evidence that Dup15q presents the opposite paradox as AS, with fast EEG oscillations typical of wakefulness during NREM sleep^18^.

As models of abnormal cortical dynamics, 15q disorders demonstrate that typical EEG patterns, such as fast EEG activity during wakefulness and slow EEG activity during NREM sleep, are not necessary for consciousness and unconsciousness, respectively. EEGs from these disorders may therefore be useful for distilling the essential common denominator across biomarkers of consciousness, thus deriving biomarkers of consciousness applicable across a broad range of dynamical regimes. Biomarkers of consciousness are most needed in situations where consciousness is dissociated from behavior^70,71^. In these situations (e.g., severe brain injury), cortical dynamics may be highly abnormal. For instance, coma patients often exhibit pathological cortical hyper-synchronization^72–74^ and post-traumatic epilepsy^75^. Training and validating classifiers on EEGs from 15q disorders addresses this challenge by removing typical EEG patterns that would otherwise guide classification of conscious state under healthy, normal conditions. While our approach is useful for distilling the essential common denominator of consciousness across a broad range of oscillatory regimes, further work will be needed to provide direct evidence that entropy features distinguish covert consciousness from unconsciousness.

Prior research using AS and Dup15q as model disorders has often focused on autism and epilepsy (e.g., see Frohlich et al.^69^). Our work departs from these prior studies by applying 15q disorders as general models of abnormal cortical dynamics that may challenge, and therefore strengthen, EEG biomarkers of conscious state. We emphasize that our findings should in no way be interpreted to mean that children with Angelman syndrome are unconscious when awake, nor than children with Dup15q are vividly dreaming throughout NREM sleep. Indeed, such an interpretation would not only be entirely unjustified given accompanying behaviors but would also undermine the very rationale for using EEG during sleep and wakefulness from these disorders to identify biomarkers of conscious state. In addition, we wish to clearly emphasize that our work herein does not directly benefit individuals with AS or Dup15q, but may instead benefit otherwise neurotypical yet unresponsive patients with severe brain injuries for whom it is exceedingly difficult to determine the patient’s level of consciousness. However, we encourage other researchers to utilize Supplementary Data 1 to test hypotheses about 15q disorders.

### Limitations and future directions

Our study only examined children due to the fact that the vast majority of EEG data collected in 15q disorders are from children and, moreover, the AS delta EEG phenotype is most pronounced at younger ages^24^. In addition, due to the low developmental abilities of children with AS and Dup15q, it was infeasible to quantitatively measure behavioral responsiveness during EEG. Future work should aim to reproduce our findings in adult participants, e.g., using pharmacological manipulations in healthy adults to reversibly induce a dissociation between consciousness and neural oscillations^33^.

In addition, several caveats of our machine learning approach should be noted. Firstly, our Dup15q validation sample size was admittedly small (*n* = 11). However, given that we also utilized a larger NT validation set (*n* = 37), we do not view this as a crucial limitation. Secondly, in the AS training set, the number of EEG datasets was larger than the number of unique participants, with multiple data for 8 AS participants spaced at least 52 weeks apart. Nonetheless, to assess generalizability, we performed both 5-fold and 10-fold cross-validation on the training set to estimate AUCs for models trained on AS data. We found very similar AUC values and other results between all approaches (Table 3, Tables S4 and S5), suggesting that our models did not overfit to the training data and indeed generalized from AS to the validation sets. In fact, unlike many machine learning studies, the validation AUC and accuracy were often greater than the training AUC and accuracy (Supplementary Table 3), likely due to the relative ease of classification using normal EEGs from participants in the NT validation set.

Finally, while our study improves on earlier work^17^ with a review of all EEG data by a neurologist to ensure correct sleep labeling, it is exceedingly difficult to score sleep from AS, and we were therefore unable to determine exact NREM stages (N1, N2, or N3) in AS. Furthermore, while we treated NREM sleep as unconscious, some conscious mentation may occur during NREM sleep^76^. However, the relative level of consciousness in NREM sleep is widely acknowledged to be much less than that of wakefulness or REM sleep^77,78^ and efforts to control for this confound in a prior study of AS^17^ found the same general pattern of EEG changes during NREM sleep as when this factor was not controlled for. Finally, while extracting REM sleep EEG might have also allowed us to examine entropy in the context of unresponsive consciousness, this was not feasible given that nearly all AS EEGs were recorded during daytime sleep that lacked sufficient REM durations.

## Conclusions

Our findings capitalize on data from rare genetic syndromes to demonstrate that EEG entropy measures of complexity successfully detect consciousness even when the spectral characteristics of neural oscillations diverge from those seen in typical conscious and unconscious states. EEG entropy measures outperformed corresponding spectral EEG measures for both individual EEG signals and EEG connectivity. Our results thus support theories that emphasize the role of complexity in consciousness and point toward future studies of neural complexity measures as markers of covert consciousness.

## Methods

### Data collection overview

To focus our analysis on the age range typically studied in 15q disorders (children and adolescents), we limited our analyses to participants aged no older than 215 months (i.e., 17 years and 11 months), thus excluding data from adults. This age range was quite broad because recruitment of narrow age ranges is infeasible in rare disorders affecting no more than 1 in 10,000 births^79,80^. Furthermore, while the chronological ages of participants were quite broad, children with AS and Dup15q display profound developmental delays. For this reason, children in these cohorts were more developmentally alike than their chronological age range would otherwise imply, e.g., no participant with AS had an expressive language age equivalent greater than 14 months (see Supplementary Table 1 and Supplementary Table 2 for developmental scores of children with AS and Dup15q, respectively). Besides excluding data from adult participants, we also excluded data from one very young participant (EEG recorded at 8 months of age) in the Dup15q cohort whose developmental abilities were similar to that of a newborn when tested at 6 months of age. Next, we discarded datasets from participants who did not have at least 15 valid windows (i.e., 15 windows of 10.9 s duration each containing no more than 20% artifact sections) in both the wake and NREM sleep condition for the 0.5 Hz Morlet wavelet transform (see EEG features below). All datasets retained had at least 1 min of usable wake or NREM sleep data; note, however, that the average data length was much longer than this (see Table 1). For a justification of this minimum data length, see Supplementary Fig. 4 and Supplementary Fig. 5. For reference, studies of early childhood development often compute spectral EEG features from children using as little as 30 s of usable data from 2 min recording given the challenges of recording EEG from young children^81^.

### AS data collection

Children with AS were recruited through an NIH funded Angelman syndrome Natural History Study [NCT00296764] and spontaneous EEG data were recorded using Xltek/Natus acquisition software at two sites (Boston Children’s Hospital and Rady Children’s Hospital San Diego) as part of the study during wakefulness and sleep in a clinical setting. Institutional review boards of Harvard Medical School and the University of California San Diego approved the study protocol. In addition, we included one child with AS whose EEG data were recorded outside of the Natural History Study as part of an overnight sleep study. Children with AS who participated in the Natural History Study were developmentally assessed using the Bayley Scales of Infant and Toddler Development, third edition (Bayley-III)^82^. Scores from this assessment can be used to quantify, among other things, behavioral responsiveness in the form expressive and receptive language.

All EEG data were acquired at one of three sampling rates: 250, 256, or 512 Hz. Some children with AS gave data at multiple visits; in these cases, we limited our analysis to data spaced at least 52 weeks apart. Periods of drowsiness and sleep were delineated by the EEG technician during recordings using data annotations, and EEG annotations were visually reviewed for quality control by a certified neurologist (coauthor M.N.), who took into account data artifacts unrelated to EEG, such as eyeblinks and muscle activity. After EEG recordings were reviewed by M.N., we excluded EEG from a 12-year-old girl with AS for whom the presence of sleep could not be confirmed. Note that because the AS EEG phenotype generally resembles slow wave sleep, sleep scoring of AS EEG into specific NREM stages (i.e., N1, N2, or N3) is often unreliable, and wake EEG can potentially be mislabeled as sleep EEG, particularly in the absence of annotations provided by an EEG technician. We therefore did not attempt to extract any particular stage of NREM sleep, though the daytime naps we recorded likely captured NREM stage 1 (N1) and stage 2 (N2) sleep. REM sleep was not obtained in daytime naps. We identified usable wake and NREM sleep in 49 EEG recordings from 37 children with AS which entered preprocessing. For further details of AS data collection, see Frohlich et al.^17^.

### NT data collection

Children referred to Massachusetts General Hospital (MGH) who tested negative for epilepsy or neurodevelopmental disorders were included as NT participants. These participants were not taking any medications acting on the central nervous system, had normal neurodevelopment, no history of events expected to alter EEG rhythms, and were born full term with a gestational age of at least 37 weeks. Children with any neurological or psychiatric diagnosis more severe than mild attentional deficits, depressive symptoms, or tics not requiring medications were excluded. Although our use of EEG data from children who were evaluated for an abnormal event at MGH biases our sample, the crucial fact for the purposes of our analysis is that all NT children had normal EEGs and could thus satisfy the requirements of a validation group with typical neural dynamics. The institutional review board of Harvard Medical School approved the study protocol. Spontaneous EEG signals were recorded from sleeping and waking states (Xltek acquisition software). All EEG data were acquired at one of four sampling rates: 200, 250, 500, or 512 Hz. Sections of clear N2 sleep during daytime naps were identified in clinical recordings by a neurophysiologist and extracted for comparison with wake sections of the same EEG recordings. As with the AS cohort, sleep was obtained during the daytime, thus precluding the possibility of extracting sufficient data from REM sleep. The duration of available N2 sleep and wake EEG varied by participant (see Results). Data from 41 NT children entered preprocessing. The raw EEG data can be accessed through the LADDER database^83^.

### Dup15q data collection

Based on the number autism spectrum disorder cases featuring this copy number variant^84^, Dup15q is estimated to have a similar prevalence as AS—roughly 1 in 10,000–24,000 births^79,80^—yet unlike AS, we did not benefit from an NIH-funded natural history study to collect a large sample of EEG; rather, children with Dup15q were recruited locally through the University of California Los Angeles (UCLA), and the institutional review board of UCLA approved the study protocol. This yielded a small yet highly valuable validation cohort. Children with Dup15q were referred through the Dup15q clinic at UCLA or recruited through the UCLA Intellectual Disability and Development Research Center (IDDRC). Overnight EEG data were recorded at the UCLA Ronald Reagan Medical Center. Developmental assessments were also administered to children at a separate visit to the UCLA Center for Autism Research and Treatment (CART) or at the Dup15q Alliance International Family Conference using the Mullen Scales of Early Learning^85^. All EEG data were acquired at a sampling rate of 200 Hz (Nihon Kohden EEG-1200 acquisition software). To find sections of suitable NREM sleep for our analysis, N2 sleep periods delineated by sleep spindles were automatically detected using the Python-based toolbox YASA (Yet Another Spindle Algorithm)^86^. The duration of available N2 sleep varied by participant (see Results). We also extracted 30-min sections of wake recordings during mid-to-late afternoon. Wakefulness was inferred by the presence of ocular (e.g., blink or saccades) and electromyogram (EMG) artifacts in data. A certified neurologist (coauthor M.N.) visually reviewed all extracted EEG sections to confirm that they were scored correctly as wakefulness or NREM sleep. After excluding data from one 8-month-old infant, EEG data from 11 children with Dup15q entered preprocessing.

### Preprocessing

All data were preprocessed in MATLAB R2019b (The MathWorks Inc., Torrance, CA, USA). We first lowpass filtered EEG signals at 45 Hz using a finite impulse response filter with the filter order selected as twice the sampling rate of the signal. Next, we highpass filtered EEG signals at 0.4 Hz using a 5th order Butterworth filter; the stopband attenuation and roll-off of this filter were optimal for attenuating drift artifacts while minimally attenuating slow oscillations ≥ 0.5 Hz (0.44 dB attenuation at 0.5 Hz). Following filtering, data were re-referenced to average to ensure that all recordings used the same reference before continuing with preprocessing. Next, we manually marked EEG sections with gross physiological and technical artifacts, which were later excluded from all analyses. Periods of flickering light stimulation intended to trigger epileptiform activity in participants with AS were also excluded. Next, we marked noisy channels to be excluded from independent component analysis (ICA) and later interpolated following data cleaning. ICA was then used (FastICA algorithm) to remove stereotyped artifacts such as EMG and eye movements^87^. Finally, we spatially interpolated noisy channels and repeated average referencing. EEG datasets were rejected if they did not yield at least 15 valid frequency transform windows for 0.5 Hz, i.e., the lowest frequency analyzed, in both the wake and sleep condition (i.e., a minimum of 39.2 s of data were required in each condition, given that the windows size for the 0.5 Hz wavelet was 10.9 s, windows overlapped by 75%, and at least 80% of the data in the window were required to be usable).

### EEG features overview

We computed spectral and entropy EEG measures using both SC and FC measures. Unless otherwise noted, SC measures were averaged across all EEG channels, as these measures generally show low spatial variability due to volume conduction. FC measures were averaged across short-range and long-range channel pairings.

### Single-channel spectral measures (scSpectral)

EEG spectral power was computed using a Morlet wavelet transform. Wavelets were logarithmically spaced (8 per octave) between 0.5 and 32 Hz. The window size for each wavelet was determined by the kernel size and the standard deviation, with lower frequencies having larger windows. In all cases, windows overlapped by 75%. Additional implementation details can be found in Frohlich et al.^17^. Elements of time-frequency representations were averaged across time to compute power spectral densities (PSDs) with units μV^2^/Hz. Next, power was integrated within each octave to compute both absolute and relative power in each of the following frequency bands (lower bounds are inclusive, upper bounds are exclusive): s (i.e., “slow” oscillations, 0.5–1 Hz), δ1 (1–2 Hz), δ2 (2–4 Hz), θ (4–8 Hz), α-σ (8–16 Hz, encompassing both alpha oscillations and sleep spindles), and β (16–32 Hz). Because EEG data were lowpass filtered at 45 Hz to attenuate muscle artifacts and line noise, it was not possible for us to examine the 32–64 Hz octave corresponding to the gamma band. Relative power was computed by normalizing the absolute power in a frequency band according to the total integrated broadband power.

### Single-channel entropy measures (scEntropy)

Entropy reflects the number of possible ways in which a sequence can be arranged and is related to how much information it contains. Though less commonly applied to EEG signals than spectral measures, entropy measures have been used in EEG analysis for decades, particularly in the context of nonlinear dynamics^88^. We utilized two approaches to estimating signal entropy: (1) modified multiscale entropy, which is based on reoccurring motifs within the signal at different timescales^89–91^, and (2) permutation entropy, which is based on unique permutations created by ordinal rankings of data^92^. These permutations depend on the temporal separation (τ) between EEG samples and the embedding dimension (m).

### Permutation entropy

The permutation entropy (PermEn) of a time series is given as

1 $${PermEn}\left(m\right)=-\frac{1}{{{{{{\rm{ln}}}}}}(m!)}\mathop{\sum }\limits_{j=1}^{m!}{p}_{j}{{{{{\rm{ln}}}}}}\left({p}_{j}\right)$$

where *m* is the number of samples considered in each permutation (i.e., the embedding dimension) and *p*_j_ is the jth permutation. PermEn was computed using code by King et al.^48^ in 5-s windows with 50% overlap after downsampling to 125 Hz with *m* = 3. Because we had not previously investigated which values of τ best separate wake and NREM sleep using PermEn in AS, we explored several different values of τ = 8, 16, 32, 64, and 128 ms (corresponding to EEG activity at 16–40 Hz, 8–20 Hz, 4–10 Hz, 2–5 Hz, and 1–2.5 Hz, respectively) without averaging across these timescales. These are the same values reported in a recent publication by Bourdillon et al.^49^, with the exception of τ = 4 ms (32–80 Hz), which we chose not to examine due to contamination of this frequency band by muscle activity in EEG data from young children. We then chose to downsample EEG to 125 Hz for this analysis based on the Supplemental Information of King et al.^48^ demonstrating that wSMI results are robust to downsampling data to this frequency.

### Modified multiscale entropy

The multiscale entropy computes sample entropy (SampEn)^89^ at multiple timescales of the signal to account for both short-term and long-term temporal patterns in the signal^91^. The SampEn is given by

2 $${{{{{\rm{SampEn}}}}}}=-{{{{{\rm{ln}}}}}}\left(\frac{\mathop{\sum }\nolimits_{i=1}^{N-m}{n}_{i}^{m+1}(r)}{\mathop{\sum }\nolimits_{i=1}^{N-m}{n}_{i}^{m}(r)}\right)$$

where *m* is the embedding dimension or length of vectors in the signal’s embedding space and ${n}_{i}^{m}(r)$ is the number of vectors ${x}^{m}({t}_{j})$ which are within a distance *r* of ${x}^{m}({t}_{i})$ without counting instances of *i* = *j* (i.e., self-matches)^89^. We implemented the modification introduced by Xie et al.^90^ which uses a sigmoidal curve, rather than a Heaviside step function, for detecting neighbors in the state space embedding of the signal to yield the modified multiscale entropy (mMSE). We used a state space radius of *r* = 0.15 of the signal’s standard deviation and dynamically updated this radius for each timescale to correct for a previously cited weakness in the original mMSE/MSE algorithm^93,94^. Computation of mMSE matched that described in the Supplemental Material of Frohlich et al.^17^, i.e., EEG data were downsampled to 200 Hz and 20 timescales were computed using 30 s nonoverlapping segments, with segments rejected if they did not include at least 100 valid samples for each timescale^95^. However, given the finding in Frohlich et al. ^17^ that only the first 10 mMSE timescales strongly participate in EEG channel-timescale clusters that differentiate NREM sleep and wakefulness in Angelman syndrome (AS)^17^, we averaged mMSE across only the first 10 timescales for our analysis and discarded those remaining (timescales 11–20).

### Lempel-Ziv complexity

The Lempel-Ziv (LZ) algorithm—sometimes referred to as Lempel-Ziv 1976 or LZ76 to distinguish it from similar compression algorithms later published by the same authors—is a general-purpose compression routine^96^. As such, it can also be used for quantifying the complexity of a sequence based on the number of unique substrings it contains. Intuitively simple sequences (e.g., sinusoids) are easier to compress due to repetition, whereas complex sequences are more difficult to compress due to a lack of repetition. Quantifying the number of unique substrings in the EEG signal requires that the signal be converted to a binary sequence. As described in the main manuscript, we binarized the EEG signal using its median value as a threshold, computed the number of distinct “patterns” (or substrings) in the signal, and then normalized the resulting number for data length by a factor N/log2(N), where N is the data length in samples.

The conventional approach of binarizing EEG data using its mean or median value prior to application of the LZ algorithm may bias the LZ entropy estimate toward that of the signal’s low frequency components^97^. In a previous publication on AS^17^, we thus utilized the generalized multiscale Lempel-Ziv (gMLZ) complexity, which was introduced by Yeh and Shi^98^ to correct for biases in the conventional approach. However, our results from this earlier publication^17^ demonstrated that delta frequency (1–4 Hz) gMLZ timescales best separated NREM sleep and wake EEG in AS, thus suggesting that the bias toward slower frequencies in the conventional approach might actually improve the accuracy of LZ as a biomarker of conscious state. Furthermore, numerous studies have successfully demonstrated that LZ relates to conscious state without utilizing a multiscale approach^5–7,38,43,44,99^. We therefore opted for the conventional LZ approach in this study, binarizing the EEG signal by its median value after first downsampling to 200 Hz. LZ was computed using 60-s nonoverlapping segments. Segments were discarded that did not contain a minimum of 2000 samples (10 s) of usable data, based on work by Gómez et al. showing the LZ stabilizes after ~2000 EEG samples^100^.

### Context tree-weighted complexity

Like LZ, the context tree-weighted (CTW) method is a general-purpose compression and prediction algorithm that can be used for a variety of data modalities, e.g., text, music, protein sequences^101^ or, in our case, symbols extracted from EEG signals. In essence, this method uses a variable-order Markov model^101^, which builds a hierarchical decomposition of the symbol string into binary decisions^102^ that can account for long-term temporal structure in the data and provide an accurate estimator of entropy rate. In practice, CTW has been found to consistently outperform LZ when compared in simulated data, in terms of higher accuracy and lower bias^103^. Here, we used a symbolic transform that discretized EEG signals into 2-quantiles using the signal median and computed the entropy rate (i.e., information per symbol) using CTW. As with LZ, 60-s nonoverlapping segments of EEG data were used to compute CTW after downsampling to 200 Hz, and segments were discarded that did not contain a minimum of 2000 samples of usable data.

### Functional connectivity overview

We computed FC according to dwPLI (a spectral measure)^104,105^ and wSMI (an entropy measure)^48^. Prior work directly comparing both measures has suggested that they are sensitive to different types of functional interactions in EEG data across wakefulness and NREM sleep^106^. For both approaches, we used 5-second 50% overlapping windows of artifact-free data. Note that we did not surface Laplacian filter EEG signals, as this is generally not advised for sparse 19-channel montages such as ours^107^. FC values were averaged separately across short-range and long-range channel pairings. Short-range pairings were defined as channel pairs with a Euclidian distance between 80–130 mm according to standardized three-dimensional Cartesian channel coordinates from an adult template, whereas long-range pairings were defined as Euclidian distances ≥130 mm.

### Functional connectivity spectral measures (fcSpectral)

The dwPLI is a fcSpectral measure that reflects the imaginary part of the cross-spectral density, which is robust to spurious phase relationships potentially arising from volume conduction, with a “debiasing” correction to avoid an inflationary bias induced by sample size^104^. Within each 5-s FC window, the cross-spectral density was computed with the MATLAB function cpsd (2-s Hamming windows with 50% overlap; frequency bins were logarithmically spaced to match wavelets from the spectral power analysis). Next, we used Fieldtrip^108^ to compute the dwPLI from the cross-spectral density and then averaged the output within the s, δ1, δ2, θ, α-σ, and β frequency bands.

### Functional connectivity entropy measures (fcEntropy)

The wSMI is an fcEntropy measure rooted in the same procedure introduced for computing PermEn. As such, it captures both temporal complexity and spatial connectivity, meaning that it can be regarded as a measure of spatiotemporal complexity. EEG signals are transformed into permutations, or sequences of discrete symbols. The joint probability of each pair of symbols is then used to compute the symbolic mutual information (SMI) between EEG channels, which is then weighted to disregard same or opposite signed symbols arising from volume condition. As with PermEn, we computed wSMI using *m* = 3 and τ = 8, 16, 32, 64, and 128 ms after downsampling data to 125 Hz. For each channel pairing, we normalized wSMI values according to local permutation entropies using the formula

3 $${wSMI}^{\prime} \left(X,Y\right)=2\left[\frac{{wSMI}\left(X,Y\right)}{{PE}\left(X\right)+{PE}\left(Y\right)}\right]$$

where PE(X) and PE(Y) are the permutation entropies of signals X and Y, respectively, and wSMI(X,Y) and wSMI’(X,Y) are the unnormalized and normalized wSMI between signal X and Y, respectively.

### Window length and total data length

To verify that we used appropriate window lengths to compute EEG entropy features, we generated 100 simulated EEG signals and studied the effect of window length on entropy estimates. EEG signals with 0 mean and unit variance were simulated using 1/f or “pink noise” with a spectral exponent of alpha = 2.0 and a simulated sampling rate of 500 Hz. We then applied the same bandpass filtering to each simulated signal as we had applied to real EEG signals. For LZ, CTW, and mMSE, simulated signals were downsampled to 200 Hz as was done for real EEG signals and entropy estimates were computed with windows sizes ranging from 2 to 100 s in 2 s increments. For PermEn, simulated signals were first downsampled to 125 Hz as was done for real EEG signals and entropy estimates were computed with window sizes ranging from 0.5 to 20 s in 0.5 s increments; this was performed separately for each value of tau (τ = 8, 16, 32, 64, and 128 ms).

Next, to examine how the entropy estimates changed as a function of window length, for each increment of window length, we computed the difference between window length n and window length *n* + 1. We then computed the mean absolute value of all such successive differences up to the current window length *n* + 1. Finally, we also computed the mean and 95% confidence intervals of this quantity across all 100 simulated signals.

Finally, to verify that the total duration of usable EEG data did not influence estimates of EEG features, we next modeled each EEG feature using LMMs with the formula:

4 $${{{{{\rm{FEATURE}}}}}} \sim {{{{{\rm{GROUP}}}}}}+{{{{{\rm{CONSCIOUS}}}}}}+{{{{{\rm{LENGTH}}}}}}+\left(1{{{{{\rm{|}}}}}}{{{{{\rm{PARTICIPANT}}}}}}\right)$$

where FEATURE is the EEG feature, GROUP is the cohort (either NT, AS, or Dup15q), CONSCIOUS is the state of consciousness (wakefulness or NREM sleep), LENGTH is the total length of usable data for each EEG recording, and (1|PARTICIPANT) indicates random intercepts for each participant. We then evaluated the significance of the term LENGTH.

### EEG peak frequencies

To characterize the spectral profile of each EEG recording, we channel-averaged and then log-scaled power spectral densities (computed with Morlet wavelets) separately for wakefulness and NREM sleep. We then used the fitting oscillations and one over f (FOOOF) algorithm^109^ with default settings to extract peaks from channel-averaged spectra, and we defined the peak frequency of each recording as the frequency of the peak with the greatest power.

### Machine learning

Having selected EEG features, we classified EEG data according to conscious state (wakefulness or NREM sleep) using RLR, selecting the regularization parameter λ after testing 100 logarithmically spaced values between 4.4 × 10^−5^ and 0.44 according to the value which gave the best fit using 10-fold cross-validation on the AS data, with NREM sleep and wake data from the same participants on the same side of the cross-validation partition. We then trained our classifier on AS data. Data from NT children and children with Dup15q were used as two separate validation sets to assess classification performance. In this way, our classifier first learned features that distinguished conscious (wakefulness) and unconscious (NREM sleep) states under conditions of abnormal cortical dynamics in AS and then assessed whether the learned features were generalizable both to healthy cortical dynamics in NT children and to a different regime of abnormal high frequency cortical activity in Dup15q.

To test the robustness of our findings to the choice of training data (see Results, Approach and Rationale), we repeated the above analyses with NT and AS switched (i.e., using NT as the training set and AS, along with Dup15q, as a validation set). Dup15q data were never used as a training set due to this cohort’s small sample size (*N* = 11). Because there were no features with sufficiently large (|β| > 0.5) regression coefficients of NREM sleep in the scSpectralA feature category of the NT data (i.e., mirroring the procedure used to select features for AS-training with LMMs would yield no features for scSpectralA), we only performed the NT-training replication using PCA feature selection. Between 2–4 PCs were used to explain at least 90% of the wakefulness—NREM sleep variance in NT data.

Finally, to further ensure that training AUCs do not reflect overfitting, we also tested the training dataset using 10-fold cross-validation for both the original analysis (AS as the training set) and the NT replication (NT as the training set), and also using 5-fold cross-validation for the original analysis. As with the hyperparameter fitting, NREM sleep and wake data from the same AS participants were kept on the same side of the cross-validation partition.

In the machine learning approach that utilized LMMs to select features, we fit LMMs to each feature in the AS data using the formula

5 $${{{{{\rm{EEG}}}}}} \sim {{{{{\rm{CONSCIOUS}}}}}}+\left(1{{{{{\rm{|}}}}}}{{{{{\rm{PARTICIPANT}}}}}}\right)$$

where EEG is the EEG feature, CONSCIOUS is a binary variable denoting wakefulness or NREM sleep, and (1|PARTICIPANT) denotes random intercepts for participants. Next, for each feature type, we selected features with |β| > 0.5 for CONSCIOUS.

For each RLR model, we assessed performance using the AUC. We also computed accuracy, precision, recall, and specificity using the optimal operating point of the ROC curve. Conscious states (i.e., wakefulness) were coded as positive classes, e.g., low specificity would indicate that a model erred toward classifying unconscious states as conscious. Although RLR models were fit to channel-averaged data for classification purposes, we also fit models to individual channel and channel-pair measures for visualization purposes. To test whether classifiers performed better than chance (i.e., AUC > 0.5), or better than each other (entropy versus spectral), we converted AUC scores to Mann–Whitney U statistics (see Statistical Analysis below). Finally, to assess the usefulness of individual features, we fit unregularized logistic regression models to AS data for each feature and validated the models using NT and Dup15q data.

### Evaluation of classifier performance

To derive 95% confidence intervals for the AUC of each ROC plot, as well as classifier accuracy, precision, recall, and specificity, we used 10^4^ bootstrapped resamples using the MATLAB function perfcurve with the bias corrected and accelerated percentile method. Next, to evaluate classifier performance, we transformed each AUC to a Mann–Whitney *U* statistic using the relation

6 $$U={{{{{\rm{AUC}}}}}}\cdot {N}^{2}$$

where *U* is the Mann–Whitney *U* test statistic and *N* is the number of participants in the balanced sample (i.e., there are *N* wake datasets and *N* sleep datasets). Next, we used an approximation of the normal distribution to covert each U to a z-score using the formula

7 $$z=\frac{U{{{{{\rm{\hbox{-}}}}}}}{N}^{2}/2}{\sqrt{\frac{{N}^{2}({N}^{2}+1)}{12}}}$$

which allows one to test the one-tailed hypothesis that the AUC is better than chance using the normal cumulative distribution function (CDF) to obtain a *P*-value from *z*. In addition, we compared *z*-scores between entropy and spectral measures to test the two-tailed hypothesis of different AUCs. A difference in *z*-scores was obtained using the formula

8 $$z^{\prime} =\frac{{z}_{1}{{{-}}}{z}_{2}}{\sqrt{\frac{1}{{N}_{1}-3}+\frac{1}{{N}_{2}-3}}}$$

where *N*_1_ and *N*_2_ are the sample sizes corresponding to *z*_1_ and *z*_2_, respectively. As before, the new z-score was used to derive a *P*-value using the normal CDF; this approach is valid as a significance test when *N*_1_ = *N*_2_, as was the case in our analysis.

### Entropy decomposition

We used an entropy decomposition to infer how much of the ΔPermEn between wakefulness and NREM sleep in AS merely reflects signal amplitude changes between these conditions, using phase-randomized surrogate data^110^. Using 1000 surrogates in all cases, we first randomized the phases within wakefulness and NREM sleep, reconstructed surrogate signals with an inverse Fourier transform, and computed PermEn to estimate the effects on PermEn of the specific pairings of phases and amplitudes in both conditions. Next, we shuffled the phases across conditions, and compared with the within-condition randomized data to infer the effects due to the phase distributions of wakefulness and NREM sleep. The difference in PermEn between wakefulness and NREM sleep data that remained after the phases had been randomized was the effect due to amplitude changes alone. Finally, phase randomization was used to infer the difference in PermEn due to a phase x amplitude interaction. See Mediano et al.^111^ for full details. Because we were primarily interested in the extent to which signal amplitude changes drive entropy changes, we added the differences in PermEn attributable to phase and interaction to create a single “non-amplitude” quantity. The number of EEG segments must be balanced between conditions for this analysis, and we therefore randomly selected 20 segments (5000 ms each) from wake and NREM sleep for each participant to be used for the entropy decomposition. Segments were drawn without replacement; however, note that segments potentially overlapped by 50% (see scEntropy above). After quantifying phase, amplitude, and interaction components in AS data, we fit LMMs with the formula

9 $$\Delta {{{{{\rm{PermEn}}}}}} \sim {{{{{\rm{COMPONENT}}}}}}+\left(1|{{{{{\rm{PARTICIPANT}}}}}}\right)$$

where ΔPermEn is the change in PermEn attributable to either amplitude or non-amplitude factors, COMPONENT is a categorical variable denoting either the amplitude or non-amplitude factor, and (1|PARTICIPANT) denotes random intercepts for participants. Note that models were fit separately for each of five values of τ for PermEn.

### Multiple comparisons correction

We controlled the FDR using the linear step up procedure by Benjamini and Hochberg^112^; this correction was performed separately to account for (1) 48 hypothesis tests in the main analysis using AS training data [i.e., 18 two-tailed tests of entropy versus spectral feature performance (Table 3) and 30 one-tailed tests of classifier performance (Supplementary Table 3)], (2) the same 18 two-tailed hypothesis tests performed again using 10-fold cross validation to report performance in the AS cohort (Supplementary Table 4), 18 two-tailed tests of entropy versus spectral features performed again using 5-fold cross validation (Supplementary Table 5), 24 hypothesis tests in the NT-training replication [i.e., 9 two-tailed tests of entropy versus spectral feature performance (Supplementary Table 6) and 15 one-tailed tests of classifier performance (Supplementary Table 7)], 42 tests of EEG data length on EEG features (Supplementary Fig. 5), and 5 hypothesis tests in the entropy decomposition (one for each PermEn timescale, Fig. 7). Note that the Benjamini-Hochberg linear-step up procedure was repeated again for all hypotheses in the analysis for instances where we tested the training set using 10-fold cross-validation.

### Reporting summary

Further information on research design is available in the Nature Portfolio Reporting Summary linked to this article.

## Supplementary information

Supplementary Material

Description of Additional Supplementary Files

Supplementary Data 1

Supplementary Data 2

Supplementary Data 3

Supplementary Data 4

Supplementary Data 5

Supplementary Data 6

Reporting Summary

## Data Availability

EEG features extracted for each participant are included in the Supplementary Data 1. The data used to generate figures in this manuscript are included in Supplementary Data (Supplementary Data 2: Fig. 2a–c; Supplementary Data 3: Figs. 2d, 3b, c, and 4; Supplementary Data 4: Fig. 3a; Supplementary Data 5, Figs. 5 and 6; Supplementary Data 6, Fig. 7). The AS EEG data used in this manuscript are available to researchers who apply for Level 2 access to the LADDER database^83^: https://www.laddertotreatment.org/for-researchers/. The consenting processes for other EEG data do not allow for them to be archived to repositories; however, they are in principle accessible upon reasonable request from the corresponding author. Note that investigators must complete a data transfer agreement with UCLA to obtain Dup15q EEG data.

## Code availability

Analysis code is publicly available at https://github.com/jfrohlich/angelman-consciousness and archived to Zenodo: 10.5281/zenodo.7428309.
