# Author Correction: Neural complexity is a common denominator of human consciousness across diverse regimes of cortical dynamics

**DOI:** 10.1038/s42003-023-04460-7

**Published:** 2023-01-13

**Authors:** Joel Frohlich, Jeffrey N. Chiang, Pedro A. M. Mediano, Mark Nespeca, Vidya Saravanapandian, Daniel Toker, John Dell’Italia, Joerg F. Hipp, Shafali S. Jeste, Catherine J. Chu, Lynne M. Bird, Martin M. Monti

**Affiliations:** 1 Department of Psychology, University of California Los Angeles, 90095 Pritzker Hall, Los Angeles, CA USA; 2 Department of Computational Medicine, University of California Los Angeles, Los Angeles, CA USA; 3 Department of Computing, Imperial College London, London, UK; 4 Department of Psychology, University of Cambridge, Cambridge, UK; 5 Department of Neurosciences, University of California San Diego, San Diego, CA USA; 6 Department of Neurology, Rady Children’s Hospital San Diego, San Diego, CA USA; 7 Center for Autism Research and Treatment, University of California Los Angeles, Semel Institute for Neuroscience, Los Angeles, CA USA; 8 Institute for Advanced Consciousness Studies, Santa Monica, CA USA; 9 Roche Pharma Research and Early Development, Neuroscience and Rare Diseases, Roche Innovation Center Basel, Basel, Switzerland; 10 Department of Neurology, Massachusetts General Hospital, Harvard Medical School, Boston, MA USA; 11 Department of Pediatrics, University of California San Diego, San Diego, CA USA; 12 Division of Genetics/Dysmorphology, Rady Children’s Hospital - San Diego, San Diego, CA USA; 13 Deptment of Neurosurgery, UCLA Brain Injury Research Center, David Geffen School of Medicine, University of California Los Angeles, Los Angeles, CA USA; 14 Present Address: Institute for Neuromodulation and Neurotechnology, University Hospital and University of Tuebingen, Tuebingen, Germany; 15 Present Address: Children’s Hospital Los Angeles, Los Angeles, CA USA

**Keywords:** Diagnostic markers, Circadian rhythms and sleep, Disorders of consciousness, Psychology

Correction to: *Communications Biology* 10.1038/s42003-022-04331-7, published online 15 December 2022.

The original version of this Article contained an error. In the first paragraph of the discussion section, text reading “... the strongest individual features were those belonging to PermEn or LRwSMI categories (Figs. 2, 5, and 6)” should instead read as “... the strongest individual features were those belonging to PermEn or LRwSMI categories (Figs. 2, 3, and 4)”.

This error has now been corrected in the PDF and HTML versions of the Article.

