# Supplementary material for: Neural complexity is a common denominator of human consciousness across diverse regimes of cortical dynamics
Source: Commun Biol. 2022 Dec 15;5:1374. doi: 10.1038/s42003-022-04331-7 (PMC9755290; doi:10.1038/s42003-022-04331-7)
Supplement: Supplementary file 1 — Supplementary Material [file 42003_2022_4331_MOESM1_ESM.pdf]

Supplementary Material
Neural complexity is a common denominator of human consciousness across diverse regimes of cortical dynamics

Joel Frohlich[1,2*], Jeffrey N. Chiang[3], Pedro A.M. Mediano[4,5], Mark Nespeca[6,7], Vidya Saravanapandian[8], Daniel Toker[1], John Dell'Italia[9], Joerg F. Hipp[10], Shafali S. Jeste[8,11], Catherine J. Chu[12], Lynne M. Bird[13,14], Martin M. Monti[1,15]

[1]Department of Psychology, University of California Los Angeles, 6513 Pritzker Hall, Los Angeles, CA, USA
[2]Institute for Neuromodulation and Neurotechnology, University Hospital and University of Tuebingen, Tuebingen, Germany
[3]Department of Computational Medicine, University of California Los Angeles, Los Angeles, CA, USA
[4]Department of Psychology, University of Cambridge, UK
[5]Department of Computing, Imperial College London, London, UK
[6]Department of Neurosciences, University of California San Diego, San Diego, CA, USA
[7]Neurology, Rady Children's Hospital San Diego, San Diego, CA, USA
[8]Center for Autism Research and Treatment, University of California Los Angeles, Semel Institute for Neuroscience, Los Angeles, CA, USA
[9]Institute for Advanced Consciousness Studies, Santa Monica, CA, USA
[10]Roche Pharma Research and Early Development, Neuroscience, Ophthalmology and Rare Diseases, Roche Innovation Center Basel, Basel, Switzerland.
[11]Children's Hospital Los Angeles, Los Angeles, CA, USA
[12]Department of Neurology, Massachusetts General Hospital, Harvard Medical School, Boston, MA, USA
[13]Department of Pediatrics, University of California San Diego, San Diego, CA, USA
[14]Genetics/Dysmorphology, Rady Children's Hospital San Diego, San Diego, CA, USA
[15]Deptment of Neurosurgery, UCLA Brain Injury Research Center, David Geffen School of Medicine, University of California Los Angeles, Los Angeles, CA, USA

Supplementary Results

Effects of data length on EEG feature estimates

For each EEG entropy measure, we observed an exponential decay curve that asymptotically approached 0 with increasing window length (Fig. S4). In other words, as the window length increased, the stability of the entropy estimate also increased, but with diminishing returns. For each entropy feature, the window size we had chosen in our EEG analysis (indicated with red vertical bars in Fig. S4) fell to the right of the inflexion point of the exponential decay curve. From this, we concluded that our choice of window length was justified in all cases.

After performing an FDR correction for each of 42 EEG features, we did not find any instances in which EEG data length significantly predicted the EEG feature estimate ($P_{FDR} > 0.3$ for all features, Fig. S5).

Machine learning classification with NT training data and AS/Dup15q validation data

For all comparisons of entropy versus spectral features using Angelman syndrome (AS) validation data, entropy features performed significantly better than spectral features (Table S6, highly significant, $p < 10^{-10}$, FDR corrected). In three of the remaining cases, AUCs were larger for entropy features, but not significantly so, and in two other cases, AUCs were saturated and thus equal (AUC = 1.0 in both cases) between entropy and spectral features. Finally, spectral features yielded a larger AUC than entropy features in only one case, but not significantly so (fcEntropy versus fcSpectral, Dup15q validation, $p = 0.83$, Table S6). Most classifiers (13 out 15) performed significantly better than chance (Table S7, $p < 0.05$, FDR corrected).

Supplementary Figures

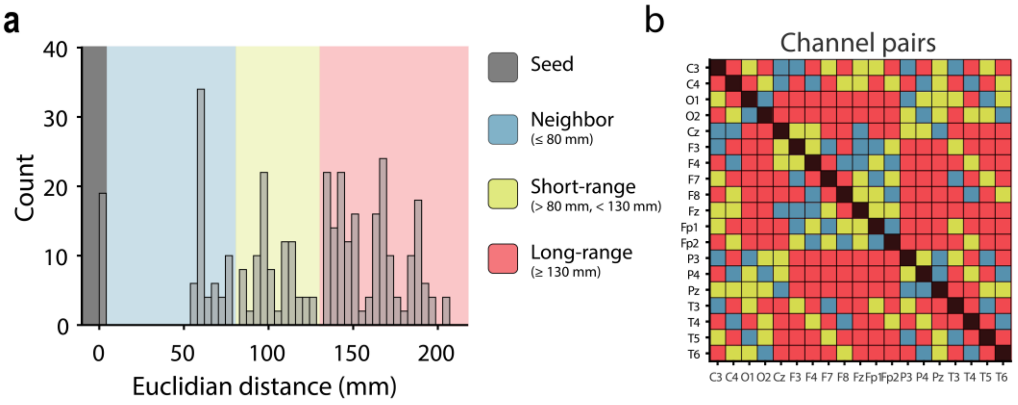

Supplementary Figure 1 Euclidian distances between 10-20 montage EEG channels. To group channels-pairings according to distance for purposes of computing functional connectivity, we examined the Euclidian distances in a standardized space between 19 channels in the 10-20 EEG montage (note that while the actual distances between channels are smaller for children as compared with the adult template standardized space, the relative distances should be preserved). (a) By examining the above histogram, we identified two gaps at approximately 80 and 130 mm. (b) Based on this distribution of channel distances, we grouped channel pairings as follows: seed (i.e., self-matches, distance = 0 mm), neighbors (distance ≤ 80 mm and > 0 mm), short- range (distances > 80 mm and < 130 mm, and long-range (distances ≥ 130 mm). Given that neighboring channels are likely to show spurious functional connectivity due to volume conduction, we only analyzed short-range and long-range connections.

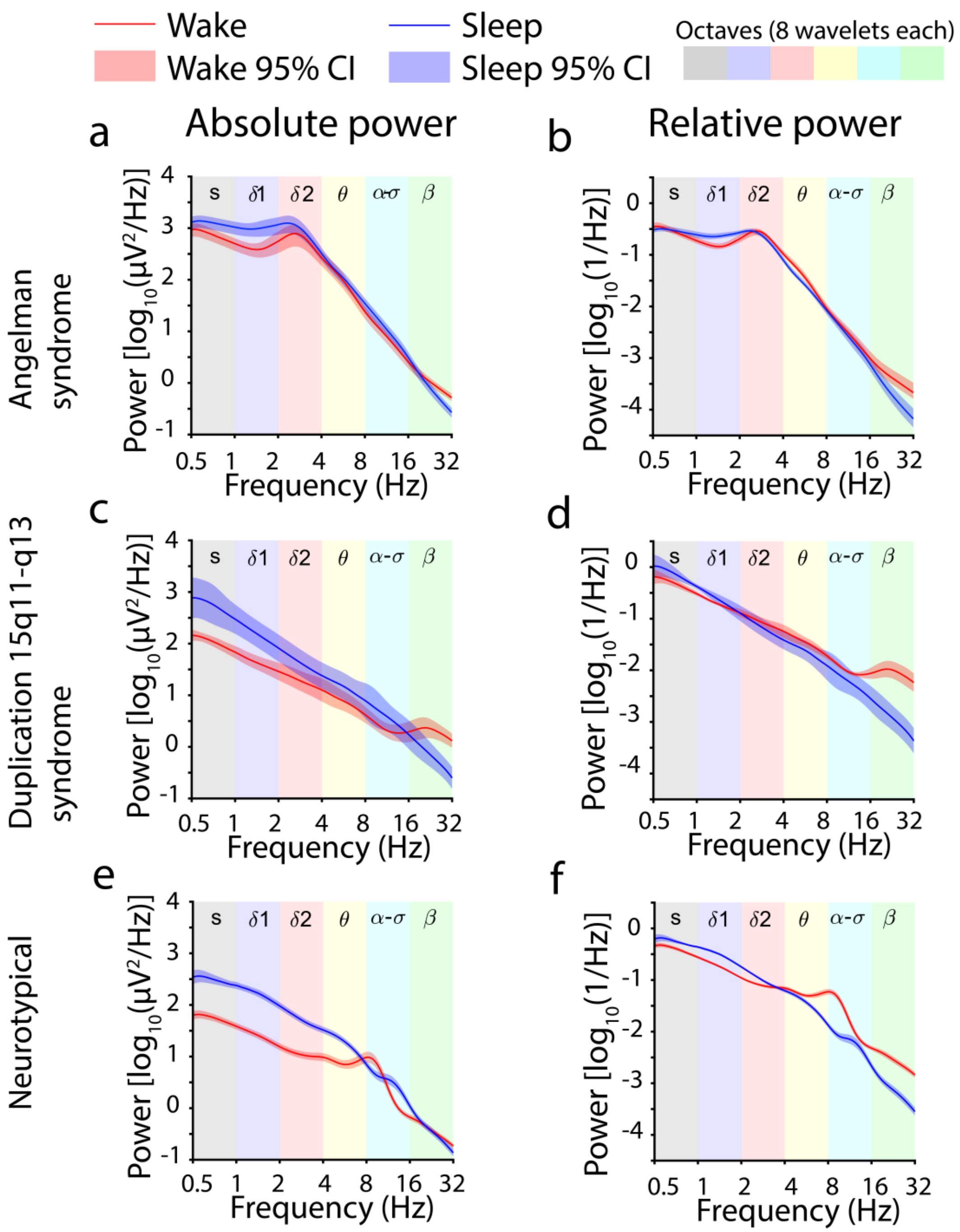

Supplementary Figure 2 Power spectral densities (PSDs) from wake and sleep. PSDs were averaged across channels and then log10-transformed in both wakefulness and NREM sleep for participants with Angelman syndrome (AS; a, b), duplication 15q11.2-13.1 syndrome (Dup15q; c, d), and neurotypical children (NT; e, f). PSDs from participants with AS were then averaged first across visits within participants with multiple visits, and then across participants.

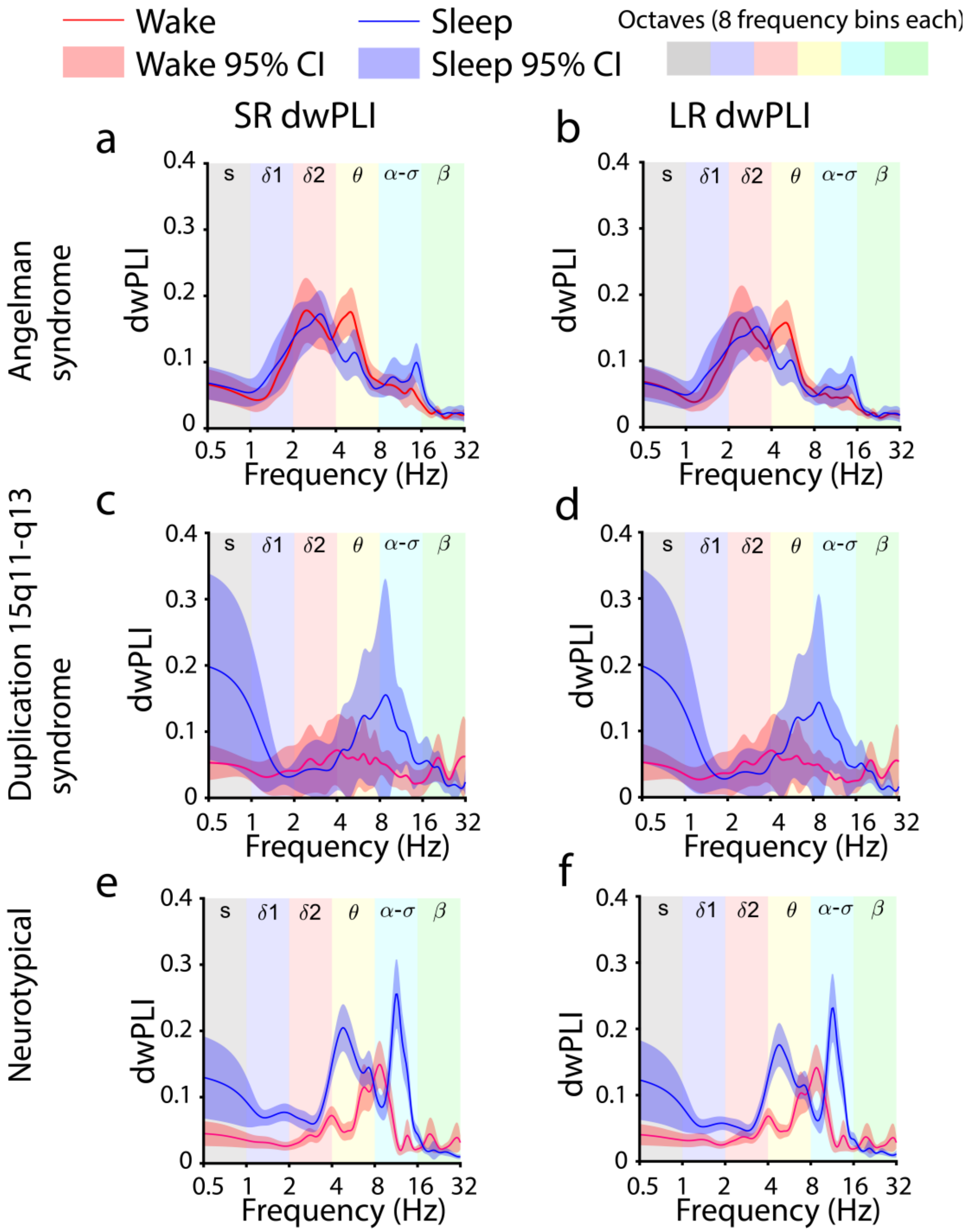

Supplementary Figure 3 Connectivity spectra measured with debiased weighted phase lag index (dwPLI). from wake and sleep. Connectivity values (dwPLI) were averaged separately across short-range and long-range electrode parings for participants with Angelman syndrome (AS; a, b), duplication 15q11.2-13.1 syndrome (dup15q; c, d), and neurotypical children (NT; e, f).

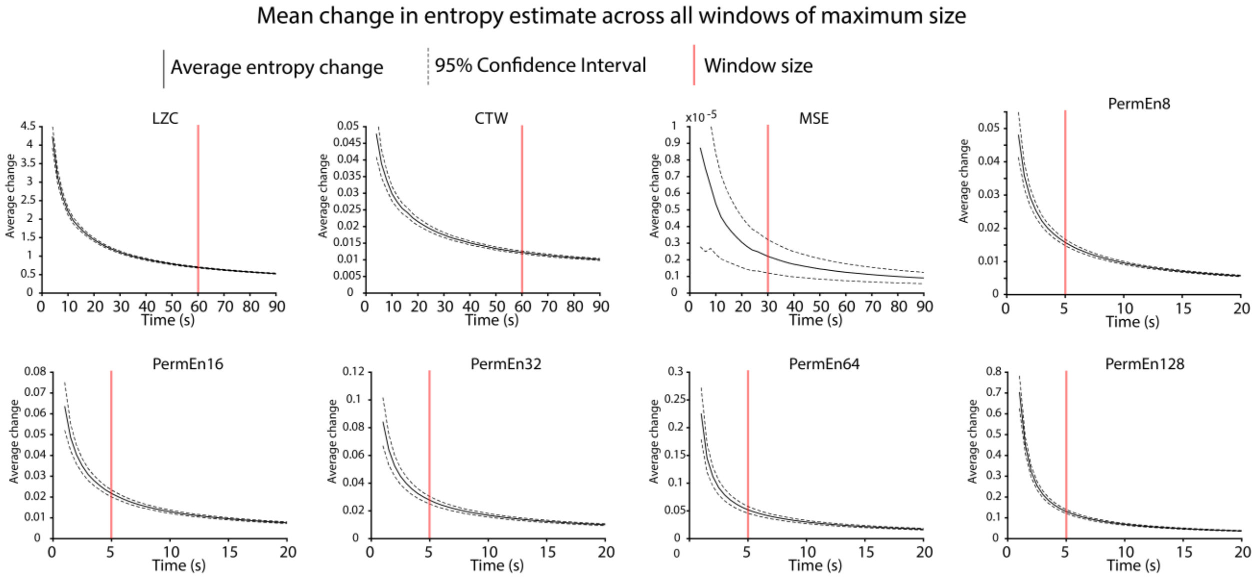

Supplementary Figure 4 Mean change in entropy estimate as a function of maximum window sizes. For each EEG entropy measure, we systematically adjusted the window size and computed the average (mean) absolute value of differences between successive window sizes; this was in turn averaged across 100 simulated signals for each entropy measure (each panel above shows the mean and 95% confidence intervals across signals). In each case, we saw an exponential decay curve (increasing stability) as a function of window length. In all cases, the window length used in our analysis (red vertical line) was beyond the inflexion point of the decay curve, indicating that we had selected a sufficient window size.

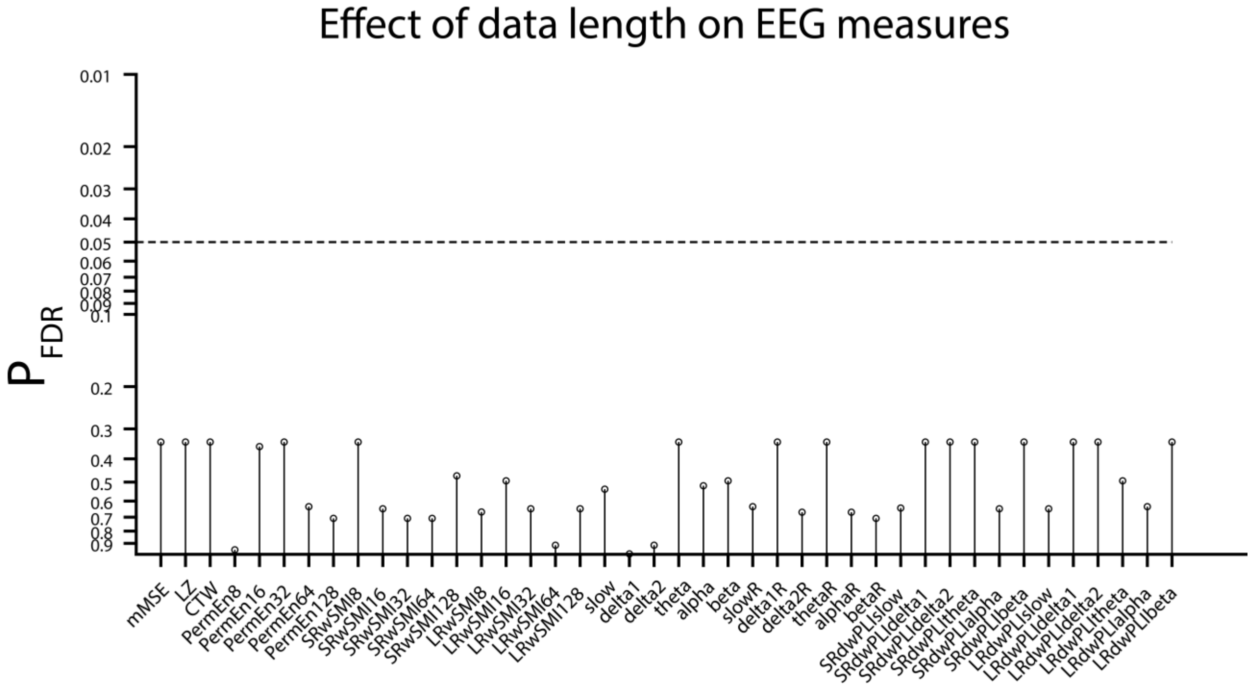

Supplementary Figure 5 EEG data length does not predict EEG feature estimates. Using linear mixed models (LMMs), we tested whether EEG feature estimates were influenced by data length while covarying for group and conscious state and also including random intercepts for participants. After evaluating the t-statistic of the term corresponding to data length in each model and applying a false discovery rate (FDR) correction for all EEG features tested, we found that EEG feature estimates could not be significantly predicted from EEG data length ($P_{FDR} > 0.3$ in all cases). The figure above depicts FDR corrected P-values for data length. Note that P-values on the vertical axis are spaced and ordered according to $-\log_{10}(P)$, i.e., smaller P-values correspond to higher tick marks. The horizontal dashed line shows the threshold for statistical significance, which was not met for any EEG feature.

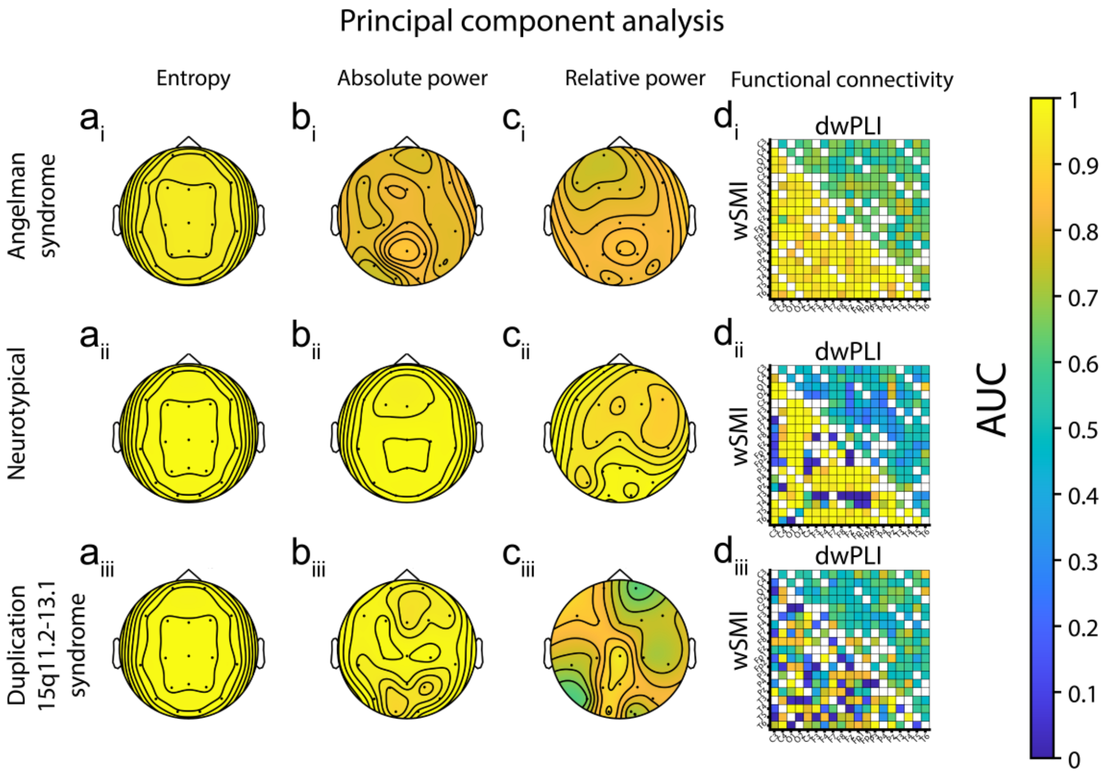

Supplementary Figure 6 Areas under curve (AUCs) for receiver operating characteristics for individual channels and channel pairs selected using principal component analysis (PCA). Features selected using PCA show largely homogenous spatial topographies for single channel (SC) entropy (a), absolute power (b), and relative power (c) features in the Angelman syndrome (AS) training set (1), neurotypical (NT) validation set (2), and duplication 15q11.2-q13.1 syndrome (Dup15q) validation set (3). The largest AUCs were observed for entropy features. Functional connectivity (FC) features were visualized by averaging PCA loadings for short-range and long-range features and applying loadings to the corresponding weighted symbolic mutual information (wSMI, entropy) and debiased weighted phase lag index (dwPLI, spectral) features for each channel pairing. Note that AUCs for wSMI are displayed in the lower triangle and AUCs for dwPLI are displayed in the upper triangle for each FC matrix; features were not computed for seed and neighboring channels, which appear white. An overall pattern of higher AUCs for wSMI, rather than dwPLI, FC features is strongly apparent.

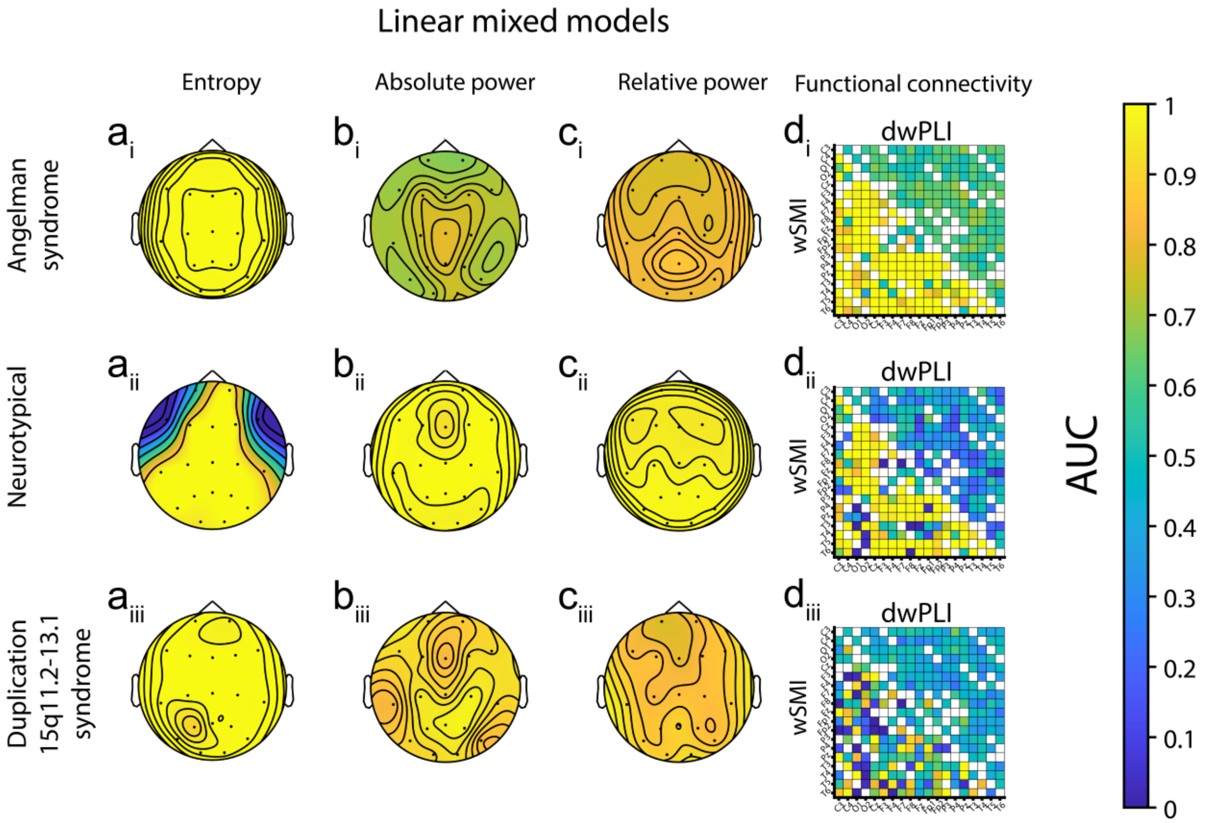

Supplementary Figure 7 Areas under curve (AUCs) for receiver operating characteristics for individual channels and channel pairs selected using linear mixed models (LMMs). Features selected using linear mixed models follow a similar pattern as that observed for PCA features. As with PCA entropy features (Supplementary Fig. 6), LMM entropy (a) features generally show perfect or near-perfect classification, with the exception of three frontal channels (F7, F8, and Fp1) in the NT validation set (a_{ii}); this local noise, possibly related to ocular artifacts, washes out in the spatial average we used to evaluate classifiers (Table 3). For visualization purposes, the AUCs of FC matrices for LMM features were computed using models fit only to the short-range features selected by LMMs (i.e., SRwSMI8 and SRdwPLIθ) for short-range channel pairings and long-range features selected by LMMs (LRwSMI8, LRwSMI16, LRwSMI32, LRwSMI64, and LRdwPLIθ) for long-range channel pairings. As with PCA FC features, a trend of greater AUCs for wSMI, rather than dwPLI, is clearly apparent for LMM FC features.

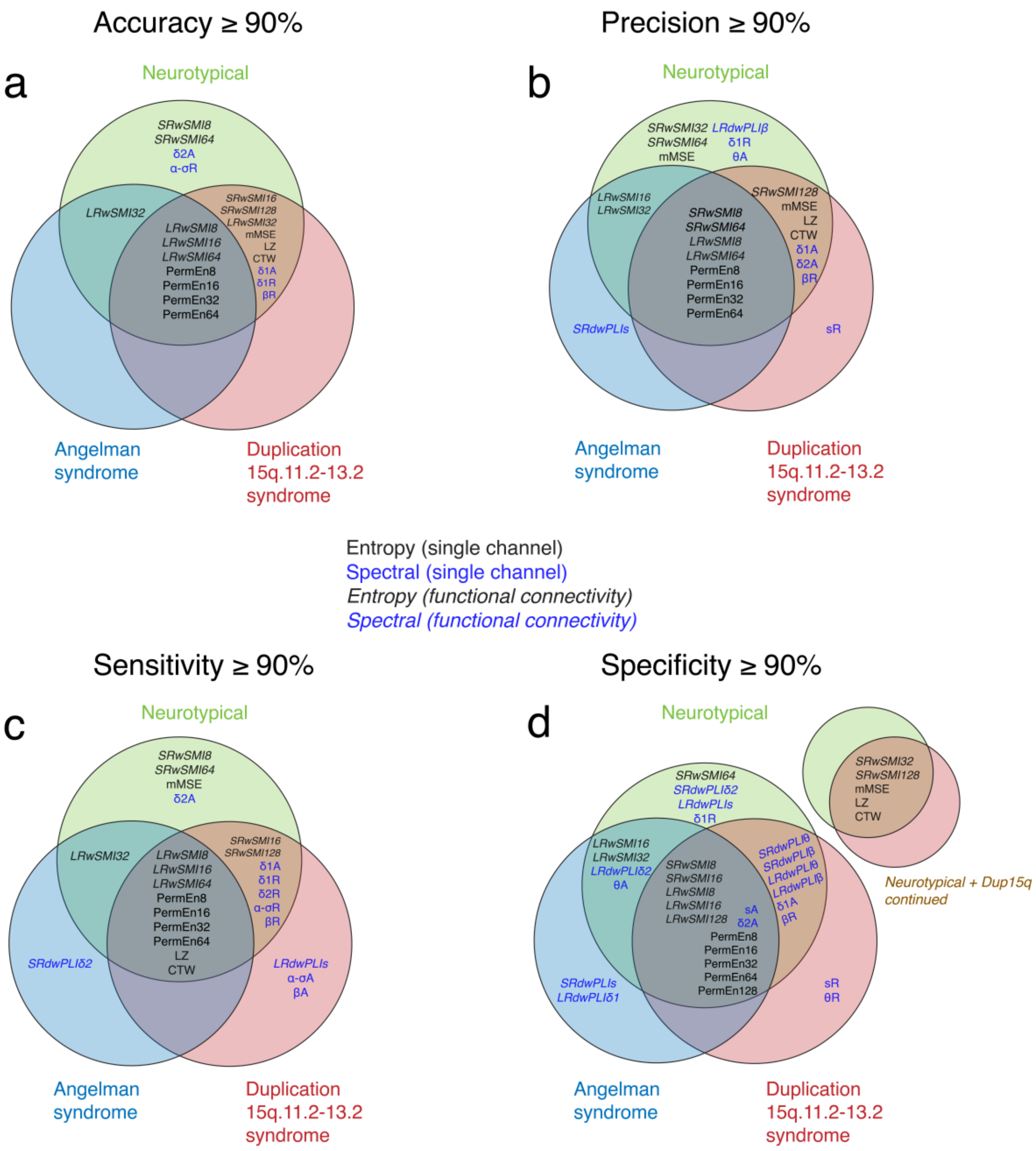

Supplementary Figure 8 Features shown inside each circle indicate EEG features with ≥ 90% performance in the corresponding dataset for other metrics: (a) accuracy, (b) precision (i.e., positive predictive value), (c) sensitivity (i.e., recall or one minus false negative rate)), and (d) specificity (i.e., true negative rate or one minus false positive rate). In (d), note that an extra pair of circles has been drawn for neurotypical and duplication 15q11.2-13.1 syndrome, as the number of features that yield ≥ 90% specificity for both cohorts is too large to depict in the main Venn diagram. As with AUC (Fig. 2), only EEG entropy features achieved ≥ 90% performance for all three datasets using accuracy, precision, and sensitivity metrics. For specificity, however, two spectral features reflecting the absolute power of low frequency oscillations (sA and δ2A) achieved ≥ 90% performance alongside 10 entropy features. Results from (c) and (d) suggest that while low

frequency oscillations in the slow and delta bands rarely mistake NREM sleep for wakeful consciousness (i.e., low false positive rate, high specificity), they are limited by low sensitivity (high false negative rate) for detecting consciousness when it is masked by low frequency activity, e.g., as it occurs in Angelman syndrome during wakeful consciousness.

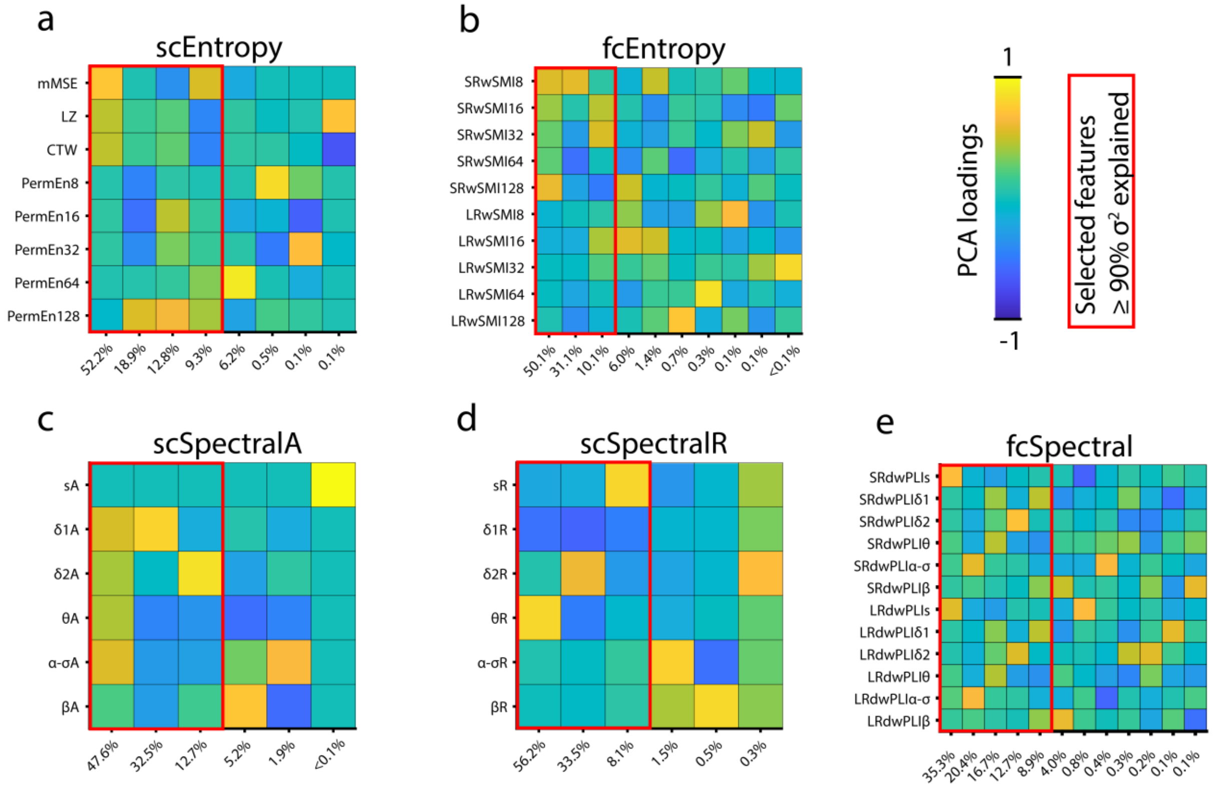

Supplementary Figure 9 Principal component analysis (PCA) loadings. We used PCA to select EEG features based on the first n features (red boxes) that cumulatively accounted for at least 90% of the variance in wake – NREM sleep features in each category for AS EEG data. (a) We selected n = 4 scEntropy PC features, which accounted for 93.2% of feature variance. The first PC was largely driven by non-permutation entropy features, whereas the second PC was largely driven by permutation entropy (PermEn) features and the remaining PCs were driven by both. (b) We selected n = 3 fcEntropy PC features, which accounted for 91.4% of feature variance. The first PC was largely driven by short-range wSMI features, and SRwSMI8 contributed very strongly to the first two PCs. (c) We selected for n = 3 scSpectralA PC features, which accounted for 92.9% of feature variance. The first PC was driven roughly evenly by δ1A, δ2A, θA, and α-σA, whereas the second PC was dominated by δ1A, and the third PC was dominated by δ2A. Slow oscillations (sA) did not contribute to any of the selected PCs, and beta oscillations (βA) contributed only mildly. (d) We selected n = 3 scSpectralR PC features, which accounted for 97.7% of feature variance. The first PC was dominated by θR, the second PC was driven almost equally by δ1R (negative loading) and δ2R (positive loading), and the third PC was dominated by sR. (e) We selected n = 5 fcSpectral PC features that accounted for 94.3% of feature variance. The first PC was dominated by beta-connectivity (SRdwPLIβ and LRdwPLIβ), while the second PC was dominated by low

delta-connectivity (SRdwPLIδ1 and LRdwPLIδ1). Across all PC features selected for fcSpectral, loadings for short-range and long-range features appeared highly correlated.

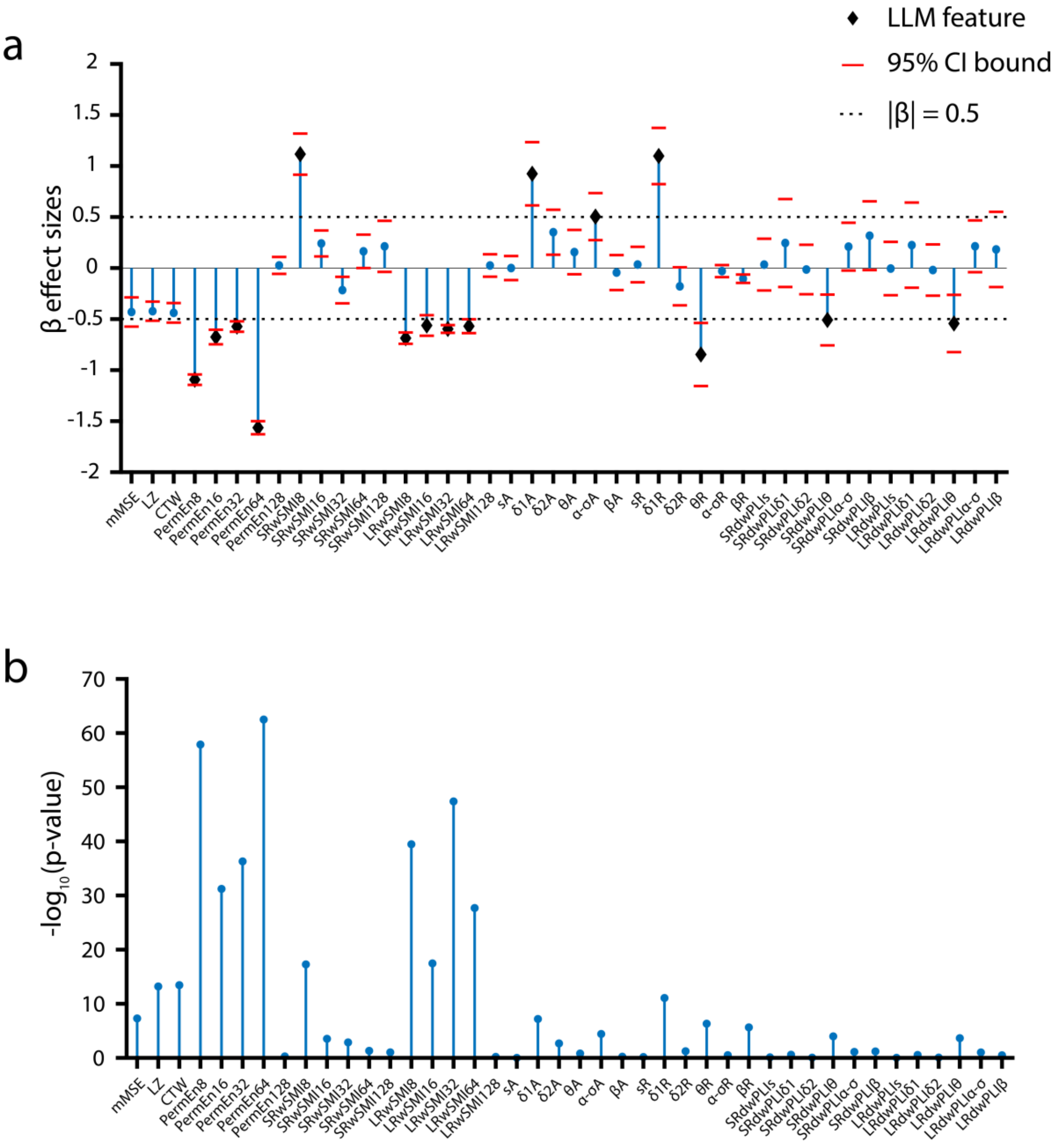

Supplementary Figure 10 Main effects of sleep in Angelman syndrome. We used linear mixed models (LMMs) to selected EEG features with large effect sizes, defined as $|\beta| \geq 0.5$ for the coefficient of the sleep term in each model. Selected features are indicated with diamonds (a), and the 95% confidence interval of β for each figure is shown using red horizontal lines. To emphasis features with strong main effects of conscious state (wake or NREM sleep), we also plotted -log10(P) for each feature (b). Note that many entropy features are highly significant, with $P < 10^{-20}$ for three weighted symbolic mutual information (wSMI) features and four permutation entropy (PermEn) features (P-values are uncorrected and were not used to make any inferences in any analysis).

Supplementary Tables

Participant	Age at EEG (months)	Days between EEG and Bayley	Receptive Language Raw Score	Receptive Language Age Equivalent	Expressive Language Raw Score	Expressive Language Age Equivalent	Fine motor Raw Score	Fine motor Age Equivalent	Gross motor Raw Score	Gross motor Age Equivalent	Cognitive Raw Score	Cognitive Age Equivalent
**9**	10	141	12	9	5	3	23	8	28	7	32	8
**6**	13	0	7	3	6	4	8	3	13	3	18	4
**2**	13	NaN	NaN	NaN	NaN	NaN	NaN	NaN	NaN	NaN	NaN	NaN
**27**	15	0	12	9	8	6	26	10	29	7	40	12
**4**	16	0	13	10	9	7	28	11	35	9	42	13
**25**	16	1	12	9	7	5	22	7	28	7	30	7
**32**	21	0	10	6	10	8	28	11	33	8	37	10
**28**	22	0	15	13	10	8	28	11	37	10	45	14
**31**	24	0	12	9	12	10	27	10	37	10	42	13
**21**	25	0	8	4	7	5	25	9	29	7	42	13
**20**	25	0	14	11	11	9	24	8	29	7	40	12
**5**	26	1	13	10	10	8	29	12	43	12	53	18
**10**	27	7	12	9	5	3	24	8	43	12	41	12
**27**	28	0	15	13	10	8	29	12	38	10	46	15
**7**	29	0	22	19	11	9	33	17	38	10	47	15
**12**	33	0	13	10	10	8	25	9	39	11	47	15
**3**	33	281	15	13	8	6	24	8	34	9	38	11
**32**	34	0	12	9	11	9	28	11	43	12	44	14
**22**	34	0	13	10	8	6	28	11	41	11	47	15
**8**	36	0	18	16	13	11	29	12	44	13	55	19
**21**	37	0	14	11	9	7	28	11	31	8	45	14
**1**	46	706	29	26	17	14	43	29	58	26	62	23
**22**	47	1	18	16	12	10	36	21	48	16	54	19
**12**	47	0	16	14	11	9	28	11	48	16	48	16

**24**	49	0	20	18	15	12	37	22	48	16	57	21
**13**	50	1	14	11	9	7	25	9	44	13	52	18
**14**	50	0	19	17	12	10	32	16	36	9	48	16
**26**	51	0	10	6	12	10	25	9	33	8	38	11
**34**	52	0	23	20	13	11	35	20	49	16	63	24
**15**	52	21	20	18	9	7	36	21	50	17	58	21
**11**	52	1	14	11	10	8	26	10	35	9	43	13
**33**	54	0	12	9	13	11	28	11	51	18	49	16
**29**	55	1	13	10	9	7	19	6	35	9	36	10
**29**	67	0	17	15	8	6	25	9	40	11	44	14
**17**	68	0	14	11	8	6	30	13	47	15	53	18
**23**	68	1	14	11	13	11	29	12	43	12	46	15
**19**	78	1	13	10	6	4	26	10	45	14	50	17
**15**	79	433	24	21	9	7	43	29	50	17	67	27
**16**	80	0	18	16	13	11	34	18	43	12	54	19
**18**	95	0	13	10	9	7	30	13	56	23	48	16
**22**	95	0	21	19	11	9	42	28	51	18	63	24
**30**	111	0	15	13	12	10	28	11	38	10	50	17
**26**	114	0	9	5	9	7	25	9	40	11	39	11

Supplementary Table 1 Developmental abilities of children with AS assessed with the Bayley Scales of Infant and Toddler Development. Participants are sorted by age, with the "Participant" column numbering matching that in Supplementary Data 1. All ages above, including age equivalents, are given in months. Developmental abilities were measured for all but one child (33/34, or 97% of participants) whose overnight EEG recording was not collected through the AS Natural History Study. Developmental assessments were usually performed the same day or within on day of the EEG recording (see columns "Days between EEG and Bayley"). In a few cases though, developmental assessments were performed much earlier or later. The absence of a flooring effect in the above data demonstrate that children with AS are behaviorally responsive during wakefulness, e.g., as demonstrated by receptive and expressive language scores. AS = Angelman syndrome; NaN = missing data (not a number).

Participant	Age at EEG (months)	Age at Mullen (months)	Receptive Language Raw Score	Receptive Language Age Equivalent	Expressive Language Raw Score	Expressive Language Age Equivalent	Visual Reception Raw Score	Visual Reception Age Equivalent	Fine Motor Raw Score	Fine Motor Age Equivalent	Gross Motor Raw Score	Gross Motor Age Equivalent
**EXCLUDED**	8	6	3	1	5	4	3	1	5	3	7	4
**40**	19	18	13	11	8	7	12	9	12	10	13	10
**43**	23	22	10	8	10	9	10	7	15	13	13	10
**45**	35	38	28	30	22	23	24	21	26	26	23	20
**44**	54	57	28	30	23	24	33	33	20	18	28	27
**39**	55	53	8	6	5	4	14	11	12	10	23	20
**41**	57	64	4	2	4	3	7	5	7	5	10	7
**42**	73	73	23	23	25	27	20	17	22	21	25	22
**37**	96	54	16	15	21	22	27	25	26	26	21	17
**38**	105	NaN	NaN	NaN	NaN	NaN	NaN	NaN	NaN	NaN	NaN	NaN
**35**	108	108	48	69	50	70	50	69	47	62	36	33
**36**	137	122	10	8	2	1	7	5	12	10	16	13

Supplementary Table 2 Developmental abilities of children with Dup15q assessed with the Mullen Scales of Early Development. Participants are sorted by age, with the "Participant" column numbering matching that in Supplementary Data 1. All ages above, including age equivalents, are given in months. Data from one participant were excluded due to young chronological and developmental age. Of the 11 children with data included in the study, developmental abilities were measured for all but one child (10/11, or 91% of participants). In all cases, assessments were performed on a separate date from overnight EEGs, usually within a few months (see columns "Age at EEG" and "Age at Mullen"). The absence of a flooring effect in the above data demonstrate that children with Dup15q are behaviorally responsive during wakefulness, e.g., as demonstrated by receptive and expressive language scores. Dup15q = Duplication 15q11.2-13.1 syndrome; NaN = missing data (not a number).

Train on	Feature selection	Feature type	Classification	Cohort	λ (regularization parameter)	P_{FDR}	Mann-Whitney U statistic	AUC (95% CI)	Accuracy (95% CI)	Precision (95% CI)	Recall (95% CI)	Specificity (95% CI)
**AS**	PCA	fcEntropy	Training	Angelman	0.005964	3.23E-12	1735	93.8% (84.4% - 98.3%)	91.9% (81.4% - 95.3%)	89.1% (81.8% - 93.3%)	95.3% (84.2% - 100.0%)	88.4% (75.0% - 95.7%)
**AS**	PCA	fcEntropy	Validation	Neurotypical	0.005964	2.33E-13	1369	100.0% (100.0% - 100.0%)	100.0% (100.0% - 100.0%)	100.0% (100.0% - 100.0%)	100.0% (100.0% - 100.0%)	100.0% (100.0% - 100.0%)
**AS**	PCA	fcEntropy	Validation	Dup15q	0.005964	0.052	87	71.9% (40.2% - 92.0%)	77.3% (31.8% - 90.9%)	75.0% (0.0% - 92.3%)	81.8% (0.0% - 100.0%)	72.7% (37.5% - 92.9%)
**AS**	PCA	fcSpectral	Training	Angelman	0.051083	6.67E-05	1381	74.7% (62.6% - 84.3%)	74.4% (55.8% - 83.7%)	72.3% (55.2% - 83.0%)	79.1% (43.2% - 96.7%)	69.8% (54.8% - 82.6%)
**AS**	PCA	fcSpectral	Validation	Neurotypical	0.051083	1	328	24.0% (14.1% - 36.3%)	51.4% (37.8% - 70.3%)	50.7% (37.7% - 65.8%)	100.0% (82.1% - 100.0%)	2.7% (0.0% - 14.7%)
**AS**	PCA	fcSpectral	Validation	Dup15q	0.051083	0.760	54	44.6% (19.6% - 70.9%)	59.1% (31.8% - 86.4%)	55.0% (28.6% - 80.0%)	100.0% (66.7% - 100.0%)	18.2% (0.0% - 54.5%)
**AS**	PCA	scEntropy	Training	Angelman	0.017994	2.61E-13	1778	96.2% (86.7% - 100.0%)	97.7% (48.8% - 100.0%)	95.6% (83.3% - 100.0%)	100.0% (27.0% - 100.0%)	95.3% (84.4% - 100.0%)
**AS**	PCA	scEntropy	Validation	Neurotypical	0.017994	2.33E-13	1369	100.0% (100.0% - 100.0%)	100.0% (100.0% - 100.0%)	100.0% (100.0% - 100.0%)	100.0% (100.0% - 100.0%)	100.0% (100.0% - 100.0%)
**AS**	PCA	scEntropy	Validation	Dup15q	0.017994	6.09E-05	121	100.0% (100.0% - 100.0%)	100.0% (100.0% - 100.0%)	100.0% (100.0% - 100.0%)	100.0% (100.0% - 100.0%)	100.0% (100.0% - 100.0%)
**AS**	PCA	scSpectralA	Training	Angelman	0.01889	1.35E-06	1484	80.3% (69.3% - 88.4%)	74.4% (64.0% - 82.6%)	71.4% (57.9% - 79.6%)	81.4% (62.7% - 97.8%)	67.4% (51.4% - 80.0%)
**AS**	PCA	scSpectralA	Validation	Neurotypical	0.01889	2.62E-10	1271	92.8% (83.4% - 97.5%)	89.2% (79.7% - 94.6%)	87.2% (77.8% - 92.3%)	91.9% (78.9% - 100.0%)	86.5% (71.4% - 95.0%)
**AS**	PCA	scSpectralA	Validation	Dup15q	0.01889	4.245E-04	113	93.4% (70.9% - 100.0%)	90.9% (63.6% - 100.0%)	84.6% (60.0% - 100.0%)	100.0% (71.4% - 100.0%)	81.8% (45.5% - 100.0%)
**AS**	PCA	scSpectralR	Training	Angelman	0.030959	1.22E-07	1538	83.2% (73.2% - 90.5%)	77.9% (64.0% - 84.9%)	75.0% (62.6% - 83.3%)	83.7% (61.7% - 97.8%)	72.1% (56.8% - 84.1%)

**AS**	PCA	scSpectralR	Validation	Neurotypical	0.030959	6.58E-13	1353	98.8% (92.9% - 100.0%)	98.6% (73.0% - 100.0%)	97.4% (96.0% - 100.0%)	100.0% (56.8% - 100.0%)	97.3% (84.7% - 100.0%)
**AS**	PCA	scSpectralR	Validation	Dup15q	0.030959	0.011	97	80.2% (49.6% - 98.3%)	86.4% (40.9% - 95.5%)	78.6% (0.0% - 94.1%)	100.0% (57.1% - 100.0%)	72.7% (37.5% - 92.9%)
**AS**	LMM	fcEntropy	Training	Angelman	0.01438	3.38E-15	1849	100.0% (100.0% - 100.0%)	100.0% (100.0% - 100.0%)	100.0% (100.0% - 100.0%)	100.0% (100.0% - 100.0%)	100.0% (100.0% - 100.0%)
**AS**	LMM	fcEntropy	Validation	Neurotypical	0.01438	2.33E-13	1369	100.0% (100.0% - 100.0%)	100.0% (100.0% - 100.0%)	100.0% (100.0% - 100.0%)	100.0% (100.0% - 100.0%)	100.0% (100.0% - 100.0%)
**AS**	LMM	fcEntropy	Validation	Dup15q	0.01438	7.645E-03	99	81.8% (45.5% - 100.0%)	90.9% (36.4% - 100.0%)	84.6% (0.0% - 100.0%)	100.0% (0.0% - 100.0%)	81.8% (45.5% - 100.0%)
**AS**	LMM	fcSpectral	Training	Angelman	0.017129	2.372E-03	1263	68.3% (55.9% - 79.0%)	67.4% (57.0% - 77.9%)	67.4% (51.6% - 77.8%)	67.4% (50.0% - 88.4%)	67.4% (52.3% - 80.6%)
**AS**	LMM	fcSpectral	Validation	Neurotypical	0.017129	1	214	15.6% (8.0% - 27.3%)	48.6% (37.8% - 59.5%)	0.0% (0.0% - 0.0%)	0.0% (0.0% - 0.0%)	97.3% (84.4% - 100.0%)
**AS**	LMM	fcSpectral	Validation	Dup15q	0.017129	0.905	46	38.0% (14.7% - 66.1%)	59.1% (36.4% - 81.8%)	75.0% (0.0% - 100.0%)	27.3% (0.0% - 70.0%)	90.9% (55.6% - 100.0%)
**AS**	LMM	scEntropy	Training	Angelman	0.000161	3.38E-15	1849	100.0% (100.0% - 100.0%)	100.0% (100.0% - 100.0%)	100.0% (100.0% - 100.0%)	100.0% (100.0% - 100.0%)	100.0% (100.0% - 100.0%)
**AS**	LMM	scEntropy	Validation	Neurotypical	0.000161	2.33E-13	1369	100.0% (100.0% - 100.0%)	100.0% (100.0% - 100.0%)	100.0% (100.0% - 100.0%)	100.0% (100.0% - 100.0%)	100.0% (100.0% - 100.0%)
**AS**	LMM	scEntropy	Validation	Dup15q	0.000161	6.09E-05	121	100.0% (100.0% - 100.0%)	100.0% (100.0% - 100.0%)	100.0% (100.0% - 100.0%)	100.0% (100.0% - 100.0%)	100.0% (100.0% - 100.0%)
**AS**	LMM	scSpectralA	Training	Angelman	0.029085	1.136E-04	1365	73.8% (62.1% - 83.2%)	70.9% (60.5% - 80.7%)	65.5% (54.5% - 77.3%)	88.4% (69.4% - 100.0%)	53.5% (38.3% - 68.3%)
**AS**	LMM	scSpectralA	Validation	Neurotypical	0.029085	2.61E-13	1366	99.8% (98.2% - 100.0%)	98.6% (91.9% - 100.0%)	100.0% (100.0% - 100.0%)	97.3% (83.0% - 100.0%)	100.0% (100.0% - 100.0%)
**AS**	LMM	scSpectralA	Validation	Dup15q	0.029085	1.235E-04	118	97.5% (82.9% - 100.0%)	95.5% (77.3% - 100.0%)	91.7% (80.0% - 100.0%)	100.0% (71.4% - 100.0%)	90.9% (53.8% - 100.0%)
**AS**	LMM	scSpectralR	Training	Angelman	0.031823	9.12E-08	1545	83.6% (73.2% - 90.7%)	77.9% (66.3% - 86.0%)	74.0% (61.5% - 82.4%)	86.0% (63.4% - 100.0%)	69.8% (54.8% - 82.1%)

**AS**	LMM	scSpectralR	Validation	Neurotypical	0.031823	2.66E-13	1365	99.7% (97.8% - 100.0%)	98.6% (90.5% - 100.0%)	97.4% (96.0% - 100.0%)	100.0% (89.4% - 100.0%)	97.3% (85.0% - 100.0%)
**AS**	LMM	scSpectralR	Validation	Dup15q	0.0241	0.00086	110	90.9% (66.2% - 100.0%)	86.4% (54.5% - 100.0%)	90.0% (50.0% - 100.0%)	81.8% (26.7% - 100.0%)	90.9% (50.0% - 100.0%)

Supplementary Table 3 Classification scores and results of one-tailed tests of better than chance performance. All P-values are FDR corrected (P_{FDR}) for multiple testing. Classification scores are expressed here as percentages, with confidence intervals (CI) derived from bootstrapping. AUC = area under ROC curve, ACC = accuracy, PPV = positive predictive value (precision), TPR = true positive rate (recall or sensitivity), TNR = true negative rate (specificity), AS = Angelman syndrome, NT = neurotypical, DS = duplication 15q11.2-13.1 syndrome.

Train on	Feature selection	Cohort	N	Test	Larger AUC	P_{FDR}	Entropy AUC	Spectral AUC
**AS**	PCA	AS	43	fcEntropy vs fcSpectral	Entropy	4.32E-52	0.923	0.708
**AS**	PCA	NT	37	fcEntropy vs fcSpectral	Entropy	2.40E-119	1.000	0.240
**AS**	PCA	Dup15q	11	fcEntropy vs fcSpectral	Entropy	2.70E-05	0.719	0.446
**AS**	PCA	AS	43	scEntropy vs scSpectralA	Entropy	9.81E-36	0.952	0.775
**AS**	PCA	NT	37	scEntropy vs scSpectralA	Entropy	2.40E-05	1.000	0.928
**AS**	PCA	Dup15q	11	scEntropy vs scSpectralA	Entropy	0.352	1.000	0.934
**AS**	PCA	AS	43	scEntropy vs scSpectralR	Entropy	4.16E-29	0.952	0.793
**AS**	PCA	NT	37	scEntropy vs scSpectralR	Entropy	0.557	1.000	0.988
**AS**	PCA	Dup15q	11	scEntropy vs scSpectralR	Entropy	0.002	1.000	0.802
**AS**	LMM	AS	43	fcEntropy vs fcSpectral	Entropy	2.78E-103	0.967	0.663
**AS**	LMM	NT	37	fcEntropy vs fcSpectral	Entropy	2.40E-119	1.000	0.156
**AS**	LMM	Dup15q	11	fcEntropy vs fcSpectral	Entropy	8.14E-12	0.818	0.380
**AS**	LMM	AS	43	scEntropy vs scSpectralA	Entropy	1.92E-93	1.000	0.711
**AS**	LMM	NT	37	scEntropy vs scSpectralA	Entropy	0.932	1.000	0.998
**AS**	LMM	Dup15q	11	scEntropy vs scSpectralA	Entropy	0.774	1.000	0.975
**AS**	LMM	AS	43	scEntropy vs scSpectralR	Entropy	2.75E-40	1.000	0.812
**AS**	LMM	NT	37	scEntropy vs scSpectralR	Entropy	0.916	1.000	0.997
**AS**	LMM	Dup15q	11	scEntropy vs scSpectralR	Entropy	0.142	1.000	0.901

Supplementary Table 4 Comparison of entropy versus spectral measures with 10-fold cross-validation used to compute Angelman syndrome (AS) areas under the receiver operating characteristic curve (AUCs). AUCs reported here for AS do not reflect training performance, but rather the performance across all 10 cross-validation folds (i.e., the classifier outputs were concatenated across all folds to generate one vector of predicted values from which a receiver operating characteristic curve was constructed). This table reports similar information as Table 3 in the main manuscript, but using 10-fold cross-validation for hyper-parameter fitting and for reporting AUCs for the AS cohort.. AS = Angelman syndrome; NT = neurotypical; DS = duplication 15q11.2-q13.1 syndrome; AUC = area under receiver operating characteristics curve; P_{FDR} = P-values corrected using false discovery rates

,

Train on	Feature selection	Cohort	N	Test	Larger AUC	P_{FDR}	Entropy AUC	Spectral AUC
**AS**	PCA	AS	43	fcEntropy vs fcSpectral	Entropy	8.69E-49	0.918334	0.710654
**AS**	PCA	NT	37	fcEntropy vs fcSpectral	Entropy	2.40E-119	1	0.319211
**AS**	PCA	Dup15q	11	fcEntropy vs fcSpectral	Entropy	0.023457	0.719008	0.570248
**AS**	PCA	AS	43	scEntropy vs scSpectralA	Entropy	3.05E-37	0.953488	0.77285
**AS**	PCA	NT	37	scEntropy vs scSpectralA	Entropy	8.75E-08	1	0.910153
**AS**	PCA	Dup15q	11	scEntropy vs scSpectralA	Entropy	0.492238	1	0.950413
**AS**	PCA	AS	43	scEntropy vs scSpectralR	Entropy	8.84E-28	0.953488	0.798269
**AS**	PCA	NT	37	scEntropy vs scSpectralR	Entropy	0.357385	1	0.983199
**AS**	PCA	Dup15q	11	scEntropy vs scSpectralR	Entropy	0.001493	1	0.793388
**AS**	LMM	AS	43	fcEntropy vs fcSpectral	Entropy	3.15E-117	0.978908	0.654949
**AS**	LMM	NT	37	fcEntropy vs fcSpectral	Entropy	2.40E-119	1	0.156318
**AS**	LMM	Dup15q	11	fcEntropy vs fcSpectral	Entropy	8.14E-12	0.818182	0.380165
**AS**	LMM	AS	43	scEntropy vs scSpectralA	Entropy	1.35E-76	0.98053	0.719308
**AS**	LMM	NT	37	scEntropy vs scSpectralA	Entropy	0.932475	1	0.997809
**AS**	LMM	Dup15q	11	scEntropy vs scSpectralA	Entropy	0.774238	1	0.975207
**AS**	LMM	AS	43	scEntropy vs scSpectralR	Entropy	1.03E-38	0.98053	0.796106
**AS**	LMM	NT	37	scEntropy vs scSpectralR	Entropy	0.915723	1	0.997078
**AS**	LMM	Dup15q	11	scEntropy vs scSpectralR	Entropy	0.182845	1	0.909091

Supplementary Table 5 Comparison of areas under the curve (AUC) of receiver operating characteristic plots for entropy versus spectral measures using 5-fold cross-validation for hyper-parameter fitting and for reporting AUCs for the AS cohort . AUCs reported here for AS do not reflect training performance, but rather the performance across all 5 cross-validation folds (i.e., the classifier outputs were concatenated across all folds to generate one vector of predicted values from which a receiver operating characteristic curve was constructed). This table reports similar information as Table 3 in the main manuscript and Table S4 above, but using 5-fold cross-validation for hyper-parameter fitting and for reporting AUCs for the AS cohort. AS = Angelman syndrome; NT = neurotypical; DS = duplication 15q11.2-q13.1 syndrome; AUC = area under receiver operating characteristics curve; P_{FDR} = P-values corrected using false discovery rates

Train on	Feature selection	Cohort	N	Test	Larger AUC	P_{FDR}	Entropy AUC	Spectral AUC
**NT**	PCA	NT	37	fcEntropy vs fcSpectral	Entropy	0.063351	1	0.967129
**NT**	PCA	AS	43	fcEntropy vs fcSpectral	Entropy	1.14E-134	0.769605	0.42239
**NT**	PCA	Dup15q	11	fcEntropy vs fcSpectral	Spectral	0.719233	0.818182	0.85124
**NT**	PCA	NT	37	scEntropy vs scSpectralA	Entropy	1	1	0.998539
**NT**	PCA	AS	43	scEntropy vs scSpectralA	Entropy	1.27E-266	0.98053	0.490806
**NT**	PCA	Dup15q	11	scEntropy vs scSpectralA	NA	1	1	1
**NT**	PCA	NT	37	scEntropy vs scSpectralR	NA	1	1	1
**NT**	PCA	AS	43	scEntropy vs scSpectralR	Entropy	1.18E-27	0.98053	0.825311
**NT**	PCA	Dup15q	11	scEntropy vs scSpectralR	Entropy	0.198082	1	0.909091

Supplementary Table 6 Comparison of areas under the curve (AUC) of receiver operating characteristic plots for entropy versus spectral measures when classifiers are trained on NT data. All P-values are FDR corrected (P_{FDR}) for multiple testing. This table presents similar information as Table 3 of the main manuscript, but with NT EEGs used as training data and AS and Dup15q EEGs used as validation data. AS = Angelman syndrome; NT = neurotypical; Dup15q = duplication 15q11.2-q13.1 syndrome.

Train on	Feature selection	Feature type	Classification	Cohort	λ (regularization parameter)	P_{FDR}	Mann-Whitney U statistic	AUC (95% CI)	Accuracy (95% CI)	Precision (95% CI)	Recall (95% CI)	Specificity (95% CI)
**NT**	PCA	fcEntropy	Training	NT	0.000403	2.33E-13	1369	100.0% (100.0% - 100.0%)	100.0% (100.0% - 100.0%)	100.0% (100.0% - 100.0%)	100.0% (100.0% - 100.0%)	100.0% (100.0% - 100.0%)
**NT**	PCA	fcEntropy	Validation	AS	0.000403	1.82E-05	1423	77.0% (64.8% - 86.6%)	81.4% (57.0% - 90.7%)	88.6% (20.0% - 94.4%)	72.1% (29.5% - 90.8%)	90.7% (78.0% - 97.5%)
**NT**	PCA	fcEntropy	Validation	Dup15q	0.000403	0.008601	99	81.8% (44.4% - 100.0%)	90.9% (36.4% - 100.0%)	84.6% (0.0% - 100.0%)	100.0% (0.0% - 100.0%)	81.8% (44.4% - 100.0%)
**NT**	PCA	fcSpectral	Training	NT	0.01825	6.30E-12	1324	96.7% (90.0% - 99.2%)	93.2% (79.7% - 98.6%)	94.4% (88.9% - 100.0%)	91.9% (64.9% - 100.0%)	94.6% (81.8% - 100.0%)
**NT**	PCA	fcSpectral	Validation	AS	0.01825	1	781	42.2% (30.0% - 55.0%)	53.5% (44.2% - 68.6%)	51.9% (41.3% - 65.3%)	93.0% (77.3% - 100.0%)	14.0% (5.5% - 27.3%)
**NT**	PCA	fcSpectral	Validation	Dup15q	0.01825	0.004207	103	85.1% (60.3% - 96.9%)	81.8% (50.0% - 90.9%)	81.8% (50.0% - 100.0%)	81.8% (33.3% - 100.0%)	81.8% (45.5% - 100.0%)
**NT**	PCA	scEntropy	Training	NT	0.000475	2.33E-13	1369	100.0% (100.0% - 100.0%)	100.0% (100.0% - 100.0%)	100.0% (100.0% - 100.0%)	100.0% (100.0% - 100.0%)	100.0% (100.0% - 100.0%)
**NT**	PCA	scEntropy	Validation	AS	0.000475	5.00E-14	1813	98.1% (89.5% - 100.0%)	98.8% (51.2% - 100.0%)	97.7% (96.8% - 100.0%)	100.0% (15.6% - 100.0%)	97.7% (87.2% - 100.0%)
**NT**	PCA	scEntropy	Validation	Dup15q	0.000475	6.56E-05	121	100.0% (100.0% - 100.0%)	100.0% (100.0% - 100.0%)	100.0% (100.0% - 100.0%)	100.0% (100.0% - 100.0%)	100.0% (100.0% - 100.0%)
**NT**	PCA	scSpectralA	Training	NT	0.00139	2.40E-13	1367	99.9% (98.8% - 100.0%)	98.6% (92.0% - 100.0%)	100.0% (100.0% - 100.0%)	97.3% (85.7% - 100.0%)	100.0% (100.0% - 100.0%)
**NT**	PCA	scSpectralA	Validation	AS	0.00139	0.705305	907.5	49.1% (37.5% - 60.4%)	53.5% (39.5% - 65.8%)	54.5% (27.8% - 71.8%)	41.9% (16.7% - 66.7%)	65.1% (50.0% - 78.2%)
**NT**	PCA	scSpectralA	Validation	Dup15q	0.00139	6.56E-05	121	100.0% (100.0% - 100.0%)	100.0% (100.0% - 100.0%)	100.0% (100.0% - 100.0%)	100.0% (100.0% - 100.0%)	100.0% (100.0% - 100.0%)
**NT**	PCA	scSpectralR	Training	NT	0.003114	2.33E-13	1369	100.0% (100.0% - 100.0%)	100.0% (100.0% - 100.0%)	100.0% (100.0% - 100.0%)	100.0% (100.0% - 100.0%)	100.0% (100.0% - 100.0%)

**NT**	PCA	scSpectralR	Validation	AS	0.003114	2.45E-07	1526	82.5% (72.0% - 89.9%)	77.9% (66.3% - 87.2%)	76.1% (62.9% - 83.9%)	81.4% (59.6% - 97.8%)	74.4% (59.6% - 86.1%)
**NT**	PCA	scSpectralR	Validation	Dup15q	0.003114	0.000988	110	90.9% (69.2% - 98.3%)	86.4% (63.6% - 100.0%)	90.0% (66.7% - 100.0%)	81.8% (46.2% - 100.0%)	90.9% (50.0% - 100.0%)

Supplementary Table 7 Classification scores and results of one-tailed tests of better than chance performance when classifiers are trained on NT data. All P-values are FDR corrected (P_{FDR}) for multiple testing. Classification scores are expressed here as percentages, with confidence intervals (CI) derived from bootstrapping. This table reports similar information as Supplementary Table 3, but with NT EEGs used as training data and AS and Dup15q EEGs used as validation data. AUC = area under ROC curve, ACC = accuracy, PPV = positive predictive value (precision), TPR = true positive rate (recall or sensitivity), TNR = true negative rate (specificity), AS = Angelman syndrome, NT = neurotypical, DS = duplication 15q11.2-13.1 syndrome.
