# Supplementary material for: Neural complexity is a common denominator of human consciousness across diverse regimes of cortical dynamics
Source: Commun Biol. 2022 Dec 15;5:1374. doi: 10.1038/s42003-022-04331-7 (PMC9755290; doi:10.1038/s42003-022-04331-7)
Supplement: Supplementary file 2 — Description of Additional Supplementary Files [file 42003_2022_4331_MOESM2_ESM.pdf]

Description of Additional Supplementary Files

File name: Supplementary Data 1
Description: EEG features.

File name: Supplementary Data 2
Description: Source data for Fig. 2a,b,c.

File name: Supplementary Data 3
Description: Source data for Fig. 2d, Fig. 3b,c, and Fig. 4.

File name: Supplementary Data 4
Description: Source data for Fig. 3a.

File name: Supplementary Data 5
Description: Source data for Fig. 5 and Fig 6.

File name: Supplementary Data 6
Description: Source data for Fig. 7.
