# Supplementary material for: Neural complexity is a common denominator of human consciousness across diverse regimes of cortical dynamics
Source: Commun Biol. 2022 Dec 15;5:1374. doi: 10.1038/s42003-022-04331-7 (PMC9755290; doi:10.1038/s42003-022-04331-7)
Supplement: Supplementary file 9 — Reporting Summary [file 42003_2022_4331_MOESM9_ESM.pdf]

nature portfolio

Corresponding author(s): Joel Frohlich

Last updated by author(s): 11/17/2022

Reporting Summary

Nature Portfolio wishes to improve the reproducibility of the work that we publish. This form provides structure for consistency and transparency in reporting. For further information on Nature Portfolio policies, see our Editorial Policies and the Editorial Policy Checklist.

Statistics

For all statistical analyses, confirm that the following items are present in the figure legend, table legend, main text, or Methods section.

n/a	Confirmed	
☐	☒	The exact sample size (n) for each experimental group/condition, given as a discrete number and unit of measurement
☐	☒	A statement on whether measurements were taken from distinct samples or whether the same sample was measured repeatedly
☐	☒	The statistical test(s) used AND whether they are one- or two-sided<br>*Only common tests should be described solely by name; describe more complex techniques in the Methods section.*
☐	☒	A description of all covariates tested
☐	☒	A description of any assumptions or corrections, such as tests of normality and adjustment for multiple comparisons
☐	☒	A full description of the statistical parameters including central tendency (e.g. means) or other basic estimates (e.g. regression coefficient) AND variation (e.g. standard deviation) or associated estimates of uncertainty (e.g. confidence intervals)
☐	☒	For null hypothesis testing, the test statistic (e.g. F, t, r) with confidence intervals, effect sizes, degrees of freedom and P value noted<br>*Give P values as exact values whenever suitable.*
☒	☐	For Bayesian analysis, information on the choice of priors and Markov chain Monte Carlo settings
☐	☒	For hierarchical and complex designs, identification of the appropriate level for tests and full reporting of outcomes
☐	☒	Estimates of effect sizes (e.g. Cohen's d, Pearson's r), indicating how they were calculated

Our web collection on statistics for biologists contains articles on many of the points above.

Software and code

Policy information about availability of computer code

Data collection	Angelman syndrome: Xltek/Natus acquisition software; neurotypical: Xltek acquisition software; Dup15q syndrome: Nihon Kohden EEG-1200 acquisition software
Data analysis	MATLAB 2019b with Fieldtrip (version dated from 20170827). Custom code will be made available at time of publication on Github: https://github.com/jfrohlich/angelman-consciousness

For manuscripts utilizing custom algorithms or software that are central to the research but not yet described in published literature, software must be made available to editors and reviewers. We strongly encourage code deposition in a community repository (e.g. GitHub). See the Nature Portfolio guidelines for submitting code & software for further information.

Data

Policy information about availability of data

All manuscripts must include a data availability statement. This statement should provide the following information, where applicable:

- Accession codes, unique identifiers, or web links for publicly available datasets
- A description of any restrictions on data availability
- For clinical datasets or third party data, please ensure that the statement adheres to our policy

EEG features extracted for each participant are included in the Supplementary Data 1. The data used to generate figures in this manuscript are included in Supplementary Data (Supplementary Data 2: Fig. 2a,b,c; Supplementary Data 3: Fig. 2d, Fig. 3b,c, and Fig. 4; Supplementary Data 4: Fig. 3a; Supplementary Data 5,

Fig. 5 and Fig 6; Supplementary Data 6, Fig. 7). The AS EEG data used in this manuscript are available to researchers who apply for Level 2 access to the LADDER repository: https://www.laddertotreatment.org/for-researchers/. The consenting processes for other EEG data do not allow for them to be archived to repositories; however, they are in principle accessible upon reasonable request from the corresponding author. Note that investigators must complete a data transfer agreement with UCLA to obtain Dup15q EEG data.

Human research participants

Policy information about studies involving human research participants and Sex and Gender in Research.

Reporting on sex and gender	We report sex ratios in Table 1. Sex was not analyzed as it was not relevant to our hypothesis about measures of consciousness.
Population characteristics	See Supplementary Data where were report this for each subject (age, sex, genotype, 15q copy-number, and diagnosis), and also see Table S1 and S2 with developmental scores.
Recruitment	Children with Angelman syndrome were recruited through an NIH funded Angelman syndrome Natural History Study [NCT00296764]; one additional child with sleep EEG was recruited outside of the Natural History Study through Rady Children's Hospital San Diego. Neurotypical children were recruited through families referred to Massachusetts General Hospital (MGH) in cases of children who tested negative for epilepsy or neurodevelopmental disorders. Children with Dup15q syndrome were recruited locally through UCLA.
Ethics oversight	Institutional review boards of the participating sites

Note that full information on the approval of the study protocol must also be provided in the manuscript.

Field-specific reporting

Please select the one below that is the best fit for your research. If you are not sure, read the appropriate sections before making your selection.

☒ Life sciences ☐ Behavioural & social sciences ☐ Ecological, evolutionary & environmental sciences

For a reference copy of the document with all sections, see nature.com/documents/nr-reporting-summary-flat.pdf

Life sciences study design

All studies must disclose on these points even when the disclosure is negative.

Sample size	Participants are children with rare disorders affecting roughly 1 in 10,000 individuals, thus we did not perform a power analysis but rather acquired data based on availability. Angelman syndrome: N = 34 participants, 43 EEGs (some gave multiple data); Neurotypical: N = 37 participants; Dup15q syndrome, N = 11 participants.
Data exclusions	In all groups, we excluded participants with persistent and/or unremovable physiological artifacts (e.g., EMG) or noise, as is standard for EEG research. Age criteria were established at the onset of our analysis (we only analyzed data from participants < 18 years of age). Angelman syndrome: we excluded participants who did not sleep, as well as participants older than 18 years of age. Neurotypical: at the time of recruitment, we screened out children with any neurological or psychiatric diagnosis more severe than mild attentional deficits, depressive symptoms, or tics not requiring medications. Dup15q syndrome: we excluded EEG from one infant participant due to very young age (EEG at 8 months) and low developmental abilities.
Replication	We verified reproducibility within the context of machine learning: 1) we trained the binary classifer on Angelman syndrome data and validated on neurotypical and Dup15q syndrome data, 2) we flipped the training and validation data and repeated the analysis with neurotypical training data and Angelman/Dup15q syndrome validation data, and 3) for the Angelman syndrome training data, we performed 10-fold cross validation.
Randomization	Not applicable, there were no treatment conditions. Each participant had EEG recordings during both sleep and wakefulness.
Blinding	No blinding, this was not relevant as there were no treatment conditions and this was a machine learning study (in this sense, the regularized logistic regression machine learning algorithm was "blinded").

Reporting for specific materials, systems and methods

We require information from authors about some types of materials, experimental systems and methods used in many studies. Here, indicate whether each material, system or method listed is relevant to your study. If you are not sure if a list item applies to your research, read the appropriate section before selecting a response.

Materials & experimental systems

n/a	Involved in the study	
☒	☐	Antibodies
☒	☐	Eukaryotic cell lines
☒	☐	Palaeontology and archaeology
☒	☐	Animals and other organisms
☐	☒	Clinical data
☒	☐	Dual use research of concern

Methods

n/a	Involved in the study	
☒	☐	ChIP-seq
☒	☐	Flow cytometry
☒	☐	MRI-based neuroimaging

Clinical data

Policy information about clinical studies

All manuscripts should comply with the ICMJE guidelines for publication of clinical research and a completed CONSORT checklist must be included with all submissions.

Clinical trial registration	https://clinicaltrials.gov/ct2/show/NCT00296764
Study protocol	See above for the Angelman syndrome Natural History Study protocol; note that our study merely used clinical data but did not share the clinical aims of the original study from which data were collected (i.e., our study is really about markers of consciousness, not 15q disorders or neurodevelopmental disorders).
Data collection	Angelman syndrome EEG data were collected at Rady Children's Hospital, San Diego and Boston Children's Hospital between 2006 and 2017. Neurotypical EEG data were collected at Harvard Massachusetts General Hospital between 2002 and 2012. Dup15q syndrome EEG data were collected at the University of California, Los Angeles (UCLA) Ronald Reagan Medical Center between 2014 and 2018.
Outcomes	N/A (this study was not a clinical trial to test any treatment or intervention, we just took existing data collected in clinical contexts to test biomarkers of consciousness)
